# Targeting the Microbiota–Metabolism–Immunity Axis: Active Components of Traditional Chinese Medicine in Colitis-Associated Colorectal Cancer

**DOI:** 10.3390/ijms27198836

**Published:** 2026-10-03

**Authors:** Qiuying Chen, Hanyi Zhang, Lei Fu, Feifei Liu, Qi Yang, Guangfa Zhang, Yaozhong Huang, Yanxi Yang, Chun Yao, Yitao Wang, Zhengcai Du, Hua Luo

**Affiliations:** 1 Engineering Research Center of Innovative Drug of Traditional Chinese and Zhuang Yao Medicine, Ministry of Education, Guangxi University of Chinese Medicine, Nanning 530200, China; chenqiuying2025@stu.gxtcmu.edu.cn (Q.C.); isylvie@foxmail.com (H.Z.); lfu@gxtcmu.edu.cn (L.F.); liufeifei2024@stu.gxtcmu.edu.cn (F.L.); zhangguangfa0826@163.com (G.Z.); 19296716019@163.com (Y.H.); m17758684254@163.com (Y.Y.); yaochun111111@163.com (C.Y.); 2 Institute of Traditional Chinese and Zhuang-Yao Ethnic Medicine, Guangxi University of Chinese Medicine, Nanning 530200, China; yangqi2023@stu.gxtcmu.edu.cn; 3 Foshan Joint Key Laboratory for the Research and Development and Industrialization of Chinese Medicinal Formulations, Guangdong Provincial Hospital of Integrated Traditional Chinese and Western Medicine, Foshan 528000, China; ytwang0726@163.com

**Keywords:** colitis-associated colorectal cancer, traditional Chinese medicine, gut microbiota, short-chain fatty acids, immune regulation

## Abstract

Colitis-associated colorectal cancer (CAC) develops in the setting of long-standing intestinal inflammation and remains a major complication of inflammatory bowel disease (IBD). Although effective control of inflammation may reduce cancer risk, approaches that specifically prevent the progression from chronic colitis to neoplasia remain limited. Disruption of the gut microbiota–metabolism–immunity axis is increasingly implicated in this process, as microbial dysbiosis and altered metabolite production can compromise epithelial barrier integrity, sustain inflammatory signaling, and disturb mucosal immune homeostasis. This review examines how active components derived from traditional Chinese medicine (TCM), particularly flavonoids, alkaloids, saponins, and polysaccharides, influence this interconnected network in CAC-related models. Rather than treating these constituents as a single group, we compare the mechanistic patterns and evidence base associated with each class. Flavonoids and alkaloids have been studied mainly in relation to inflammatory signaling and epithelial barrier protection, whereas saponins are more often linked to regulation of the immune microenvironment. Polysaccharides have been studied most frequently in relation to microbiota remodeling, short-chain fatty acid production, and mucosal immune balance. These distinctions are not absolute, and substantial overlap exists across component classes. Moreover, most evidence is derived from animal or cell-based studies, with marked variation in experimental models, compound characterizations, dosing, and outcome assessments. The microbiota–metabolism–immunity axis provides a coherent framework for interpreting the multi-level actions of TCM-derived components, but their causal mechanisms and clinical relevance to human CAC require further validation.

## 1. Introduction

Colorectal cancer (CRC) remains a leading cause of cancer-related morbidity and mortality worldwide, and its global burden is projected to increase further over the coming decades [1]. Colitis-associated colorectal cancer (CAC) is a distinct subtype of CRC that arises in the context of chronic intestinal inflammation, particularly in patients with inflammatory bowel disease (IBD), including ulcerative colitis (UC) and, to a lesser extent, Crohn’s disease (CD). Unlike sporadic CRC, CAC generally follows an inflammation–dysplasia–carcinoma sequence characterized by recurrent epithelial injury, defective mucosal repair, immune dysregulation, microbial dysbiosis, and inflammation-driven molecular alterations [2,3]. Additional molecular changes, including miRNA-mediated dysregulation of inflammation-related pathways, may also contribute to UC-associated carcinogenesis [4]. 

Current first-line therapies for IBD, including aminosalicylates, glucocorticoids, immunomodulators, and biologics, can effectively control intestinal inflammation in many patients. Although sustained control of inflammatory activity may reduce CAC risk, these treatments do not completely prevent inflammation-associated neoplastic progression [2]. Their long-term use may also be limited by adverse effects, primary or secondary non-response, and relapse after treatment withdrawal [3,5,6]. Accordingly, effective and well-tolerated strategies capable of targeting multiple drivers of inflammation-associated tumorigenesis are still needed.

The gut microbiota has emerged as a central regulator of CAC pathogenesis [7]. Often described as the “second human genome”, the intestinal microbiome interacts closely with host metabolism, mucosal immunity, and epithelial barrier integrity [4,8]. Under homeostatic conditions, commensal microorganisms metabolize dietary and host-derived substrates into bioactive products, including short-chain fatty acids (SCFAs) [9], secondary bile acids, and tryptophan-derived indoles [10,11]. These metabolites regulate epithelial repair, inflammasome activity, regulatory T-cell differentiation, Th17 responses, and tumor-promoting inflammation. SCFAs, in particular, are major mediators of microbiota–immune communication and contribute to maintenance of barrier integrity and immune homeostasis [10,12,13].

In CAC, dysbiosis disrupts this microbiota–metabolism–immunity network. Common alterations include reduced microbial diversity, depletion of SCFA-producing taxa such as *Roseburia*, *Faecalibacterium*, and *Butyricicoccus*, and expansion of potentially pathogenic bacteria, including *Fusobacterium nucleatum* and adherent-invasive *Escherichia* coli [11,14]. Reduced SCFA availability compromises epithelial barrier integrity and facilitates the translocation of lipopolysaccharides and other microbial products, thereby sustaining TLR4/NF-κB signaling and associated STAT3- and NLRP3-dependent inflammatory programs [15].

Other microbial metabolites further shape host responses. Microbial transformation of primary bile acids generates diverse secondary bile acids that exert context-dependent effects on inflammation and tumorigenesis, partly through FXR- and TGR5-related signaling [16]. Tryptophan-derived indoles activate the aryl hydrocarbon receptor (AhR) to support epithelial barrier integrity and mucosal immune homeostasis [17]. Perturbation of these metabolic pathways may therefore accelerate the progression from chronic colitis to CRC.

Traditional Chinese medicine (TCM) has a long history of use in gastrointestinal disorders. Accumulating preclinical evidence, together with emerging clinical observations in IBD, suggests that TCM may have therapeutic potential in inflammation-associated intestinal tumorigenesis [18]. TCM-derived bioactive components—including flavonoids, alkaloids, saponins, and polysaccharides—often act on multiple targets, a property potentially suited to the multifactorial pathogenesis of CAC [19]. Representative constituents such as berberine, baicalin, ginsenosides, and Ganoderma lucidum polysaccharides have been reported to suppress pathogenic bacteria, enrich beneficial or SCFA-producing taxa, restore SCFA and bile acid homeostasis, inhibit NF-κB/STAT3/NLRP3 signaling, and rebalance Th17/Treg responses [16,17,20,21]. Through these coordinated actions, TCM-derived active components may interrupt the mutually reinforcing interactions among dysbiosis, metabolic disturbance, immune dysfunction, and tumorigenesis.

Recent evidence identifies the gut microbiota as an important mediator of the effects of Chinese herbal medicines on CRC through the regulation of microbial composition and metabolism, mucosal immunity, and the biotransformation of herbal constituents [22]. However, existing reviews have mainly discussed CRC as a whole, whereas evidence specific to CAC and to distinct classes of TCM-derived active components remains scattered. In addition, many mechanistic findings are derived from chemically induced animal models, experimental colitis models, sporadic colorectal cancer models, or in vitro experiments, and their relevance to human CAC has not been fully established.

Accordingly, this review compares four major classes of TCM-derived active components—flavonoids, alkaloids, saponins, and polysaccharides—within the microbiota–metabolism–immunity framework. The comparison is structured around a common set of mechanistic domains, including gut microbial composition, microbial metabolites, epithelial barrier function, inflammatory signaling, and innate and adaptive immune responses. Where possible, evidence obtained directly from CAC models is distinguished from findings in experimental colitis, sporadic colorectal cancer, non-intestinal models, and cell-based systems. Examining the four classes within this shared framework allows us to identify mechanisms that recur across component classes, as well as areas in which research has concentrated on particular types of constituents. Because the available studies differ substantially in model design, intervention, and outcome assessment, the purpose of this comparison is not to rank efficacy, but to clarify current mechanistic patterns, differences in the strength of CAC-specific evidence, and priorities for further investigation.

## 2. Class-Specific Regulation of the Microbiota–Metabolism–Immunity Axis by TCM-Derived Active Components

### 2.1. Flavonoids

Flavonoids are polyphenolic secondary metabolites widely distributed in medicinal plants such as *Scutellaria baicalensis* Georgi, *Glycyrrhiza uralensis* Fisch., and *Pueraria lobata* Ohwi. Owing to their specific chemical structures, most flavonoids exhibit low oral bioavailability and poor absorption in the upper gastrointestinal tract. This pharmacokinetic property enables substantial colonic accumulation, which may facilitate bidirectional crosstalk between flavonoids and the gut microbiota [19]. On one hand, gut microbiota can metabolize flavonoids into potentially more bioactive derivatives (e.g., converting baicalin into baicalein); on the other hand, flavonoids may reshape gut microbial composition and promote the production of beneficial metabolites such as SCFAs, thereby modulating the intestinal immune microenvironment and potentially interfering with the transition from colitis to cancer [23]. Preclinical evidence supporting these effects is summarized below.

#### Mechanism Summary: From Dysbiosis to Immune Regulation

**Figure 1:**
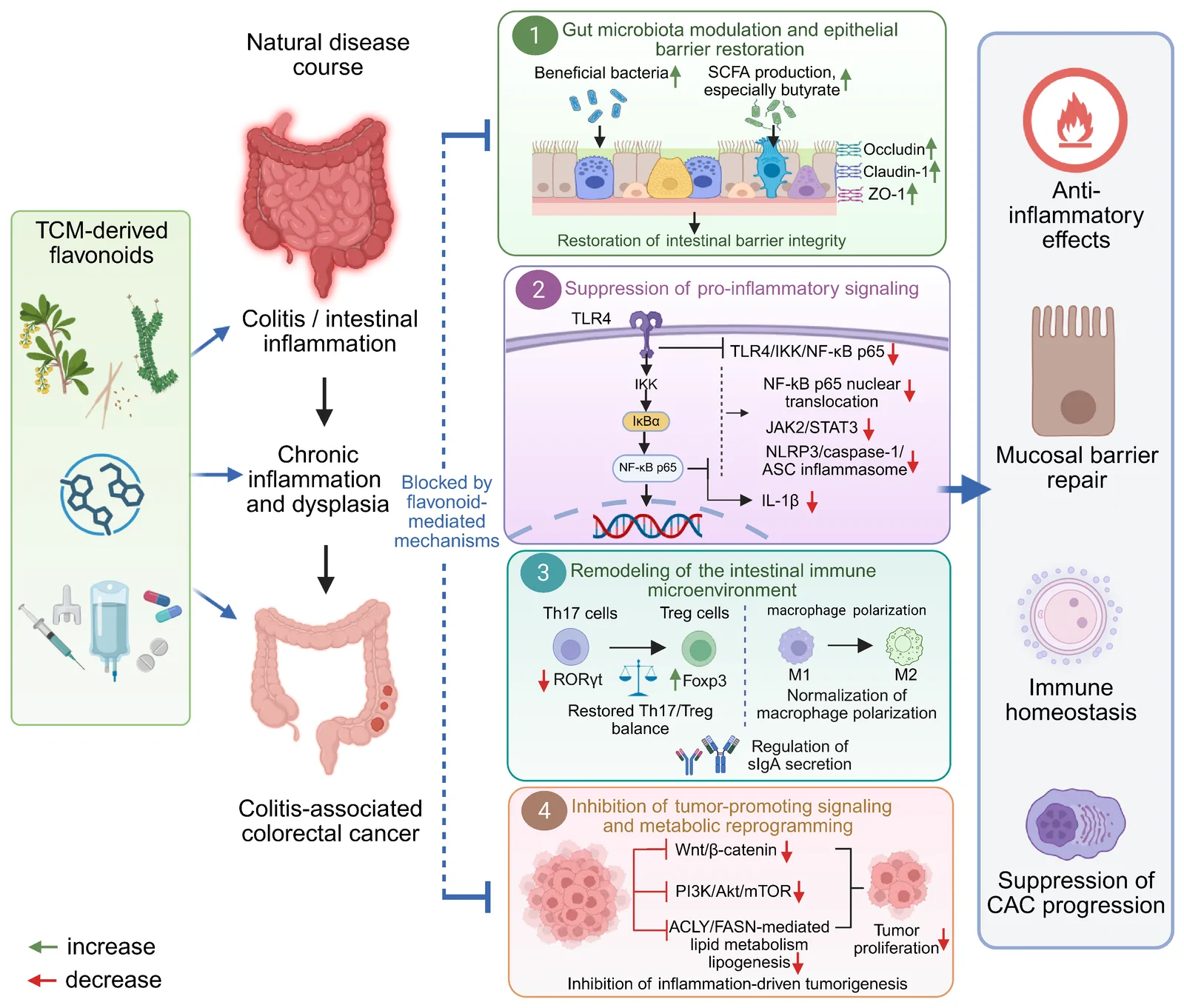
Summary of flavonoids in CAC-related models through the microbiota–metabolism–immunity axis. TCM-derived flavonoids are associated with modulation of the gut microbiota and epithelial barrier, attenuation of NF-κB, JAK2/STAT3, and NLRP3 pro-inflammatory signaling, changes in the immune microenvironment, and regulation of Wnt/β-catenin, PI3K/Akt/mTOR, and lipid metabolism-related oncogenic pathways, thereby influencing CAC-related initiation and progression.

Accumulating preclinical evidence suggests that flavonoids may exert their anti-CAC effects partly by reversing dysbiosis and enhancing metabolite-mediated barrier protection, as illustrated in Figure 1.

Microbial regulation: In experimental models, flavonoids have been reported to reverse the depletion of beneficial bacteria and inhibit the expansion of pathogenic bacteria. For example, baicalin, the dominant flavonoid in *Scutellaria baicalensis*, has been shown to increase the abundance of SCFA-producing bacteria including *Butyricicoccus, Roseburia* and *Eubacterium*, while reducing the level of *Proteobacteria* in TNBS-induced colitis [24]. Luteolin has also been reported to restore intestinal flora diversity in rats with UC, increasing the abundance of *Lactobacillus* and *Bifidobacterium* [25]. Baicalin has additionally been associated with an increasing firmicutes/bacteroides (F/B) ratio, which may contribute to an improvement in the intestinal microenvironment. Notably, these observations are derived from colitis models rather than classical CAC models; the direct relevance of these findings to CAC requires further validation in dedicated CAC models.

Metabolite-mediated barrier and immune regulation: The restoration of SCFA-producing bacteria by flavonoids has been associated with elevated colonic levels of acetate, propionate and butyrate, which are key mediators of the microbiota–metabolism–immunity axis. For example, baicalin has been reported to increase butyrate concentrations, promote Treg cell differentiation and suppress Th17 cell hyperactivation, thereby restoring Th17/Treg homeostasis in experimental settings [26]. Mechanistically, this immune modulation is often linked to inhibition of inflammatory signaling pathways. Oroxylin A has been shown to maintain colonic mucus barrier integrity and reduce colitis susceptibility by reshaping the intestinal microbiota under dietary fiber-deficient conditions, blocking NF-κB cascade activation, and decreasing IL-1β and IL-6 secretion [27]. Quercetin has been reported to alleviate UC by regulating the intestinal flora and tryptophan metabolism, promoting the production of AhR ligands (indole derivatives), and enhancing intestinal barrier function through upregulating ZO-1 and Occludin [28]. Collectively, these findings suggest that the anti-CAC potential of flavonoids may be largely attributed to prebiotic-like effects: selectively enriching butyrate-producing bacteria (e.g., *Roseburia*, *Butyricicoccus*) to elevate SCFA levels, strengthen intestinal barrier integrity, and inhibit NF-κB/STAT3-driven inflammatory responses. However, since the majority of these data is derived from colitis or in vitro models rather than CAC models, these mechanisms should be regarded as working hypotheses requiring further experimental verification.

Representative studies on TCM-derived flavonoids in CAC- or colitis-related models, including experimental models, microbiota/metabolic alterations, and key mechanisms, are summarized in Supplementary Table S1 [24,26,29,30,31,32,33,34,35,36].

### 2.2. Alkaloids

**Figure 2:**
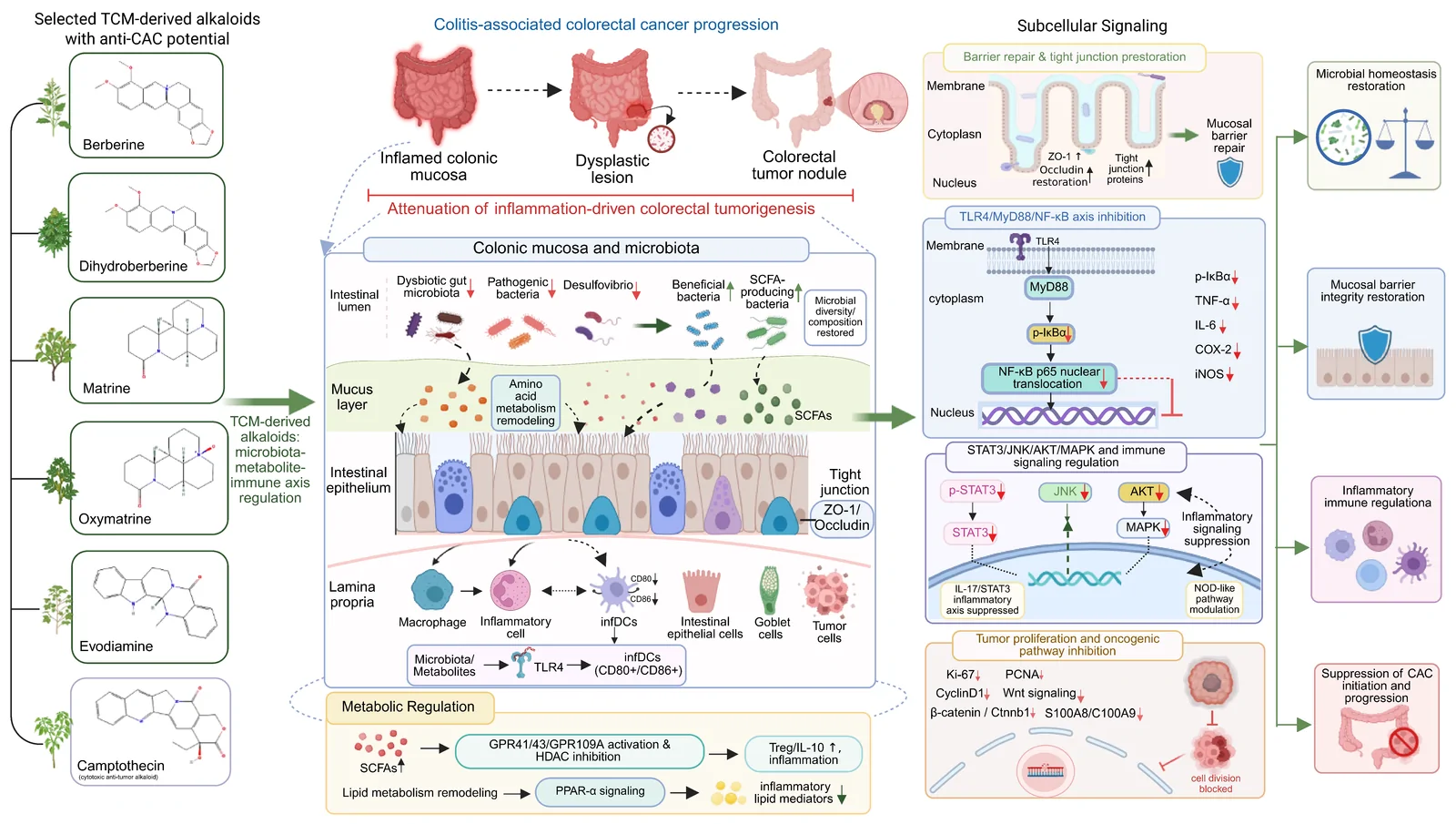
Summary of alkaloids in CAC-related models through the microbiota–metabolism–immunity axis. TCM-derived alkaloids support mucosal barrier and tight junction integrity, with potential inhibition of TLR4/MyD88/NF-κB and STAT3/JNK/AKT/MAPK signaling, and might interfere with tumor-proliferative pathways, which together might restrict CAC initiation and progression.

Alkaloids are nitrogen-containing organic compounds widely distributed in Chinese herbal medicines including *Coptis chinensis*, *Sophora flavescens* and *Evodia rutaecarpa*. Among these, berberine is the most extensively investigated alkaloid in the context of CAC. Despite its extremely low oral bioavailability (<1%), berberine has demonstrated gut-targeted anti-inflammatory and anti-tumor effects in preclinical models, suggesting the gut microbiota as a primary molecular target [37]. The representative TCM-derived active components in alkaloids with potential relevance to CAC and their microbiota–metabolism–immunity axis-mediated mechanisms are summarized in Figure 2.

#### 2.2.1. Pathogen Suppression and Barrier Restoration

Alkaloids have been reported to exert core functions by directly inhibiting opportunistic pathogens and restoring intestinal barrier integrity via metabolite-mediated signaling. In the AOM/DSS-induced CAC mouse model, berberine treatment significantly reduced the abundance of tumor-promoting bacteria including *Fusobacterium nucleatum* (*F. nucleatum*) and *Peptostreptococcus*, while enriching beneficial bacteria such as *Akkermansia muciniphila* and *Bifidobacterium* [38]. Matrine, a key alkaloid from *Sophora flavescens*, has been shown to remodel the gut microbiota by enriching *Barnesiella* and suppressing inflammation-associated *Helicobacter* [39]. Evodiamine has been reported to increase *Lactobacillus acidophilus* abundance and enhance acetate production, thereby ameliorating UC in experimental models [40]. These observations, while promising, are predominantly derived from preclinical studies and should not be interpreted as established clinical efficacy.

#### 2.2.2. Metabolite-Mediated Barrier and Immune Modulation

Berberine has been reported to restore SCFA levels (notably butyrate) and modulate bile acid metabolism. Elevated butyrate subsequently inhibits the TLR4/MyD88/NF-κB signaling cascade, reduces pro-inflammatory cytokine expression (TNF-α, IL-6), and upregulates tight junction proteins (ZO-1, Occludin), thereby promoting intestinal barrier repair in experimental settings [41]. Oxymatrine has similarly been reported to ameliorate colitis via microbiota modulation and TLR/NF-κB pathway inhibition, a mechanism linked to the regulation of inflammatory dendritic cells (DCs) [42]. Sinophylline has been shown to mitigate DSS-induced colonic injury in mice by remodeling the gut microbiota (e.g., increasing the F/B ratio) and suppressing the NF-κB and HO-1/Nrf2 pathways [43]. The unique mechanistic feature of alkaloids (particularly berberine) appears to be their dual capacity to directly suppress CAC-associated pathogens (e.g., *F. nucleatum*) and promote barrier-repairing commensals (e.g., *Akkermansia muciniphila*). Their potential anti-CAC efficacy is hypothesized to rely heavily on the microbiota–SCFA–barrier axis to interrupt the inflammation-to-cancer transition. However, these effects have been characterized primarily in animal models and in vitro systems; whether they translate to clinical benefit in CAC patients remains to be established.

Representative studies on TCM-derived flavonoids in CAC- or colitis-related models, including experimental models, microbiota/metabolic alterations, and key mechanisms, are summarized in Supplementary Table S2 [38,39,40,42,44,45,46,47,48].

### 2.3. Saponins

Saponins are a class of glycoside compounds widely present in Chinese herbal medicines (including *Panax ginseng*, *Astragalus membranaceus*, *Glycyrrhiza uralensis* and *Bupleurum chinense*), consisting of aglycones (triterpenoids or steroids) conjugated with sugar chains [49]. Regarding pharmacokinetic characteristics, saponins usually exhibit extremely low oral bioavailability (<5%) due to their large molecular weight, strong water solubility and high polarity [50]. This “pharmacokinetic drawback” may translate into a colon-directed distribution pattern: unabsorbed saponins reach the colon in their prototype form, where they may serve as fermentable substrates for intestinal flora or undergo enzymatic hydrolysis to generate secondary metabolites with altered bioactivity [51]. This bidirectional “drug–microbe activation” interaction renders saponins candidate molecules for further investigation in CAC intervention [52].

Notably, the structural similarity between ginsenosides and bile acid skeletons has been proposed to facilitate their binding to FXR and TGR5, thereby potentially regulating the microbiota–bile acid–immunity axis [53], a regulatory effect far less prominent for flavonoids and alkaloids. This hypothesis, while mechanistically plausible, requires direct experimental validation in CAC-specific models.

#### From Dual “Microbiota–Bile Acid” Axis Regulation to Immune Microenvironment Remodeling

Detailed information on representative TCM-derived saponins, their experimental models, molecular targets, and microbiota-related effects is provided in Supplementary Table S3 [54,55,56,57,58,59,60,61,62,63,64,65,66,67].

Comparing between flavonoids (which exert prebiotic-like effects to enrich SCFA-producing bacteria) and alkaloids (which may directly suppress pathogenic bacteria), the potential anti-CAC mechanism of saponins has been proposed to involve a “dual metabolic channel” mode: simultaneously elevating SCFA levels and modulating bile acid metabolism, which may in turn trigger downstream immune regulatory effects. The following evidence, unless otherwise specified, is derived from preclinical studies and should be interpreted as mechanistic hypotheses rather than clinically validated effects.

In ApcMin/+ colon cancer mice, triterpenoid saponins have been reported to reduce the abundance of sulfate-reducing bacteria (e.g., *Desulfovibrio*) and enrich beneficial commensals (including *Akkermansia*), with associated suppression of the intestinal inflammatory microenvironment [54]. Astragaloside IV has been reported to elevate the abundance of *Lactobacillus* and *Faecalibacterium*, accompanied by enhancing colonic SCFA levels [68]. While these observations are mechanistically informative, the specific contribution of SCFA elevation to the observed anti-tumor effects has not been definitively established through loss-of-function or intervention experiments.

Ginsenoside (Compound K) has been reported to remodel intestinal microbiota composition to regulate bile acid-metabolizing bacteria, modulate bile acid homeostasis via the FXR signaling pathway, and enhance the 7α-hydroxylation activity of gut flora toward primary bile acids, thereby inhibiting excessive generation of deoxycholic acid and increasing the UDCA/DCA ratio [53,69,70]. This synergistic “SCFA–bile acid” dual metabolite model has been proposed as a distinctive feature of saponins in repairing metabolic homeostasis disruption during inflammation-to-cancer transformation; however, that direct evidence linking these bile acid modulations to CAC prevention in vivo remains incomplete.

At the innate immune level, astragaloside has been reported to promote the repolarization of tumor-associated macrophages from the pro-tumor M2 phenotype toward the anti-tumor M1 phenotype (characterized by elevated iNOS and CD86, reduced Arg-1 and CD206) by inhibiting macrophage AMPK signaling, while suppressing NLRP3 inflammasome activation and reducing IL-1α release [71,72]. Anemapasin AIII has been reported to alleviate intestinal inflammation in DSS-induced colitis mice by inhibiting the TLR4-NF-κB/MAPK signaling pathway and restoring the Th17/Treg immune balance [73]. At the adaptive immunity level, ginsenoside Rg3 has been reported to suppress colorectal cancer growth by downregulating the C/EBPβ/NF-κB signaling pathway [55] and to ameliorate inflammatory disorders by reducing the expression of core pro-inflammatory cytokines (IL-6, TNF-α, IL-17, IL-1β), inhibiting NF-κB/NLRP3 inflammasome signaling and rebalancing Th17/Treg subsets [74].

**Figure 3:**
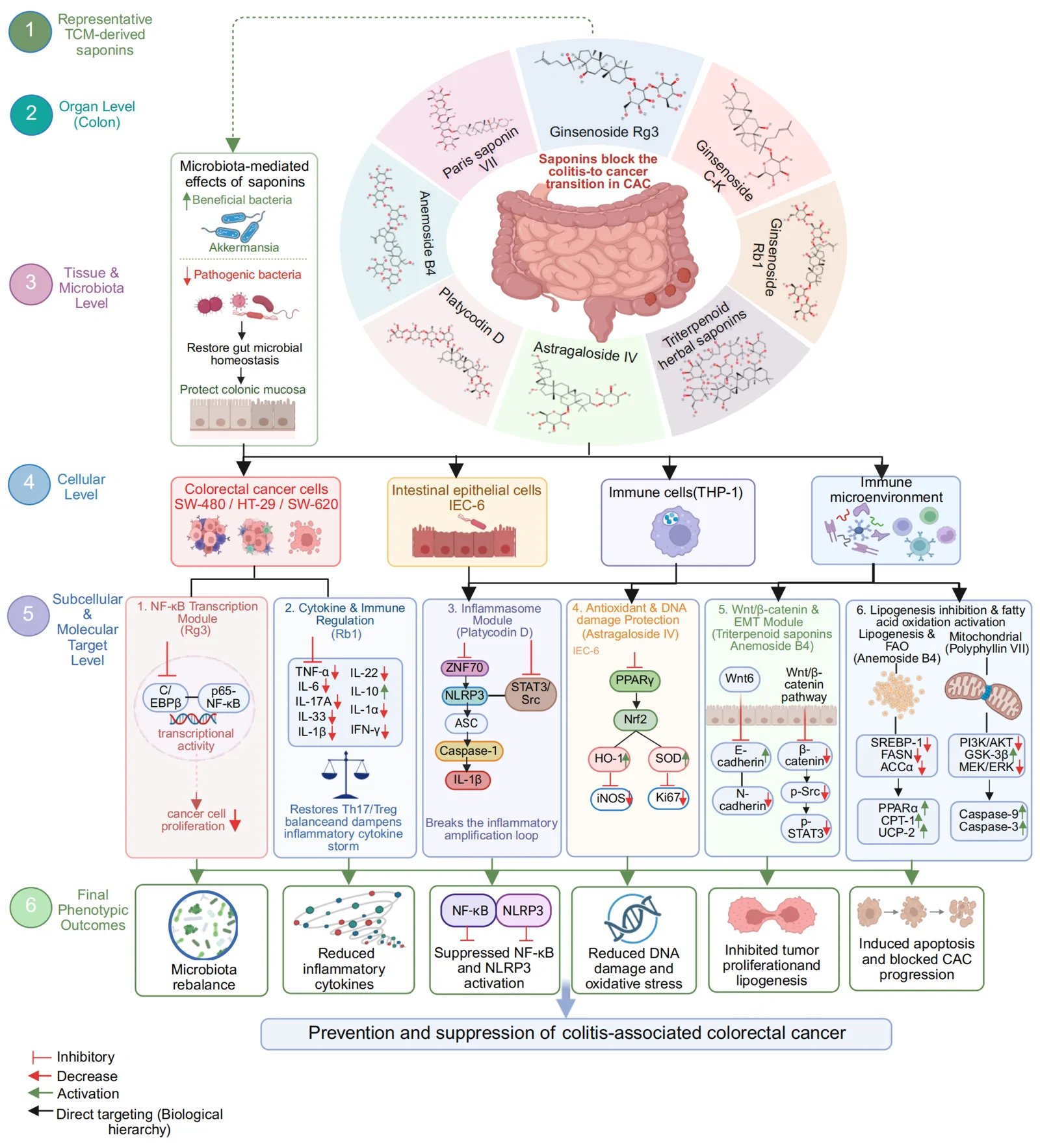
Summary of saponins in CAC-related models through the microbiota–metabolism–immunity axis. TCM-derived saponins may restore gut microbial homeostasis, protect colonic mucosa, attenuate NF-κB, NLRP3, Wnt/β-catenin, and lipogenic signaling, modulate immune balance, and induce tumor cell apoptosis, potentially blocking the colitis-to-cancer transition.

Within the microbiota–metabolism–immunity axis, the postulated anti-CAC mechanism of saponins may involve a multi-level regulatory cascade: (1) activation of a synergistic “SCFA + bile acid” dual metabolic pathway via modulation of microbial bile acid transformation; (2) dual barrier repair potentially mediated by butyrate/HDAC inhibition-associated transcriptional regulation and UDCA/TGR5 activation-mediated secretion promotion; and (3) immune microenvironment remodeling (from macrophage repolarization to Th17/Treg rebalancing) via multi-point blockade of signaling cascades such as TLR4-NF-κB. This multi-level, multi-target closed-loop regulatory mechanism positions saponins as pivotal active ingredients of TCM for CAC prevention and treatment, as summarized in Figure 3. These mechanistic proposals are supported by convergent preclinical observations, but none have been confirmed in clinical CAC settings, and the causal relationships among these events remain to be established.

### 2.4. Polysaccharides

Polysaccharides are high-molecular-weight carbohydrates polymerized from multiple monosaccharide units via glycosidic bonds, widely distributed in TCM and edible fungi, including *Astragalus membranaceus*, *Ganoderma lucidum*, *Lycium barbarum*, *Poria cocos* and other medicinal fungi. Unlike small-molecule monomers (flavonoids, alkaloids, saponins), polysaccharides possess a large molecular weight (typically >10 kDa) and lack corresponding transmembrane transporters, rendering them barely absorbable in the upper gastrointestinal tract after oral administration; instead, they are believed to reach the colonic lumen in their intact form [75,76]. This unique pharmacokinetic property may endow polysaccharides with a distinctive niche in CAC prevention and treatment. As natural fermentable substrates for intestinal flora, polysaccharides may be gradually degraded by polysaccharide lyases secreted by *Bacteroides*, *Bifidobacterium* and other commensal bacteria, ultimately generating SCFAs via anaerobic fermentation [77,78]. If the dual metabolic channel of saponins is defined as a midstream axis intervention, polysaccharides may act as upstream substrate drivers to initiate positive feedback remodeling of the entire microbiota–metabolism–immunity axis.

#### From “Substrate-Driven” to Immune-Microenvironment-Level Remodeling

Gut dysbiosis during CAC progression has been characterized not only by depleted commensals, enriched pathogens and impaired microbial metabolic function, but also by compromised SCFA synthetic capacity [79,80]. Polysaccharides have been proposed to reconstruct the growth dominance of beneficial bacteria through trophic competition and ecological niche remodeling. However, the causal role of polysaccharide-driven microbiota changes in mediating anti-CAC efficacy has not been rigorously established; most studies to date report correlational observations rather than proof of causality.

Astragalus polysaccharide (APS), among the most extensively studied components in this field, has been reported in DSS-induced colitis mice to modulate the gut microbiota, enrich SCFA-producing bacteria, restore intestinal SCFA synthesis and secretion, and activate the SCFA-FFAR2/FFAR3 signaling pathway, thereby suppressing inflammation and re-establishing immune homeostasis [81]. Ganoderma lucidum polysaccharides (GLPs) have been reported in AOM/DSS-induced CAC mice to preserve the intestinal barrier by increasing goblet cell count, promoting MUC2 mucus secretion and upregulating tight junction proteins (ZO-1, Occludin), while also enriching beneficial genera including *Lactobacillus* and *Bifidobacterium* [82]. Poria cocos polysaccharides (PCPs) have been reported to enhance intestinal barrier function and reduce permeability in AOM/DSS-induced CAC mice by restoring the secretion of tight junction proteins (E-cadherin, ZO-1, Claudin-3) and MUC2 [83]. Hericium erinaceus polysaccharide (HEP) has been reported to regulate intestinal mucosal immunity in mice, upregulating small intestinal mucosal SIgA secretion and activating intestinal MAPK and AKT signaling pathways [84]. While these findings provide supportive evidence, the majority is derived from single studies with relatively small sample sizes.

At the innate immune level, butyrate has been proposed to inhibit HDAC activity and activate GPR43/GPR109A receptors to suppress transcriptional release of pro-inflammatory cytokines (TNF-α, IL-6, IL-1β) and drive macrophage polarization toward the anti-inflammatory M2 phenotype [23]. GLP has been reported to modulate gut flora and immune cell function to attenuate colonic inflammation and tumorigenesis in colitis and CAC models via modulating macrophage activity and reducing pro-inflammatory factor levels [82]. At the adaptive immune level, atractylodes macrocephala polysaccharides (AMPs) have been reported to rebalance Th17/Treg subsets in DSS-induced colitis mice by inhibiting the IL-6/STAT3 pathway [85]. Lentinan, an a-β-1,3-glucan with a unique triple helical conformation, has been reported to exhibit a distinctive immune activation pattern: recognizing and binding to the pattern recognition receptor Dectin-1 on macrophage surface, triggering immune crosstalk between macrophages and type III innate lymphocytes (ILC3s), activating the IL-22 signaling pathway, promoting damaged intestinal mucosal repair, and enhancing intestinal antibacterial defense in experimental UC models [86]. Notably, most of these findings are derived from colitis models rather than CAC models, and whether polysaccharide-mediated immune modulation translates to CAC prevention or therapy remains to be determined.

**Figure 4:**
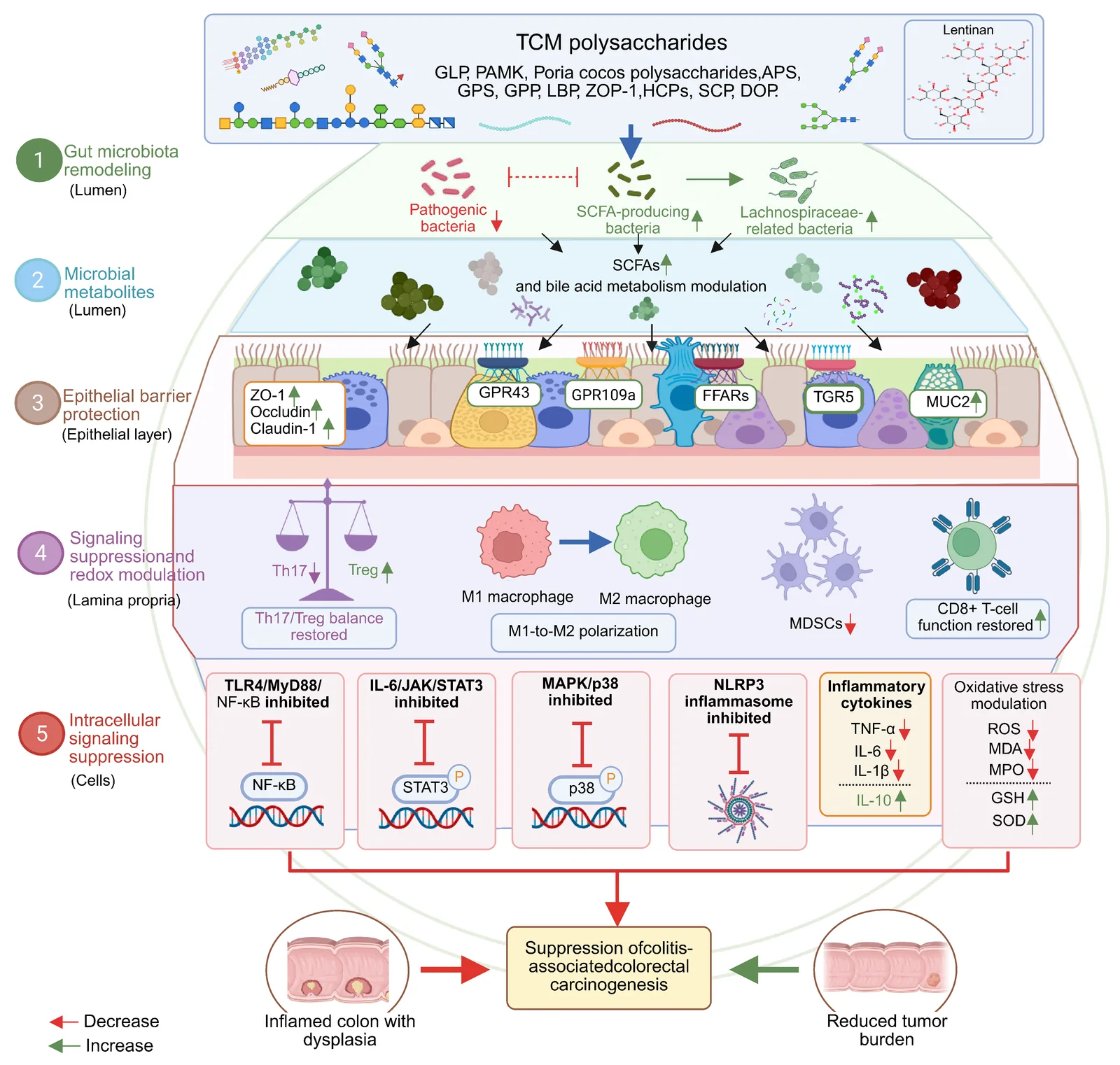
Summary of polysaccharides in CAC-related models through the microbiota–metabolism–immunity axis. TCM-derived polysaccharides might regulate the composition of gut microbiota, affect the metabolism of short-chain fatty acids and bile acids, and strengthen the integrity of the epithelial barrier to some extent. It also has a regulatory effect on NF-κB, STAT3, MAPK, and NLRP3 signaling pathways. These multiple pathways may help restore immune and redox balance, potentially playing a role in slowing down the progression of colitis-related cancer.

The proposed anti-CAC mechanism of polysaccharides centers on a “substrate-driving hierarchical amplification” model along the microbiota–metabolism–immunity axis. (1) As natural prebiotic substrates, polysaccharides may selectively enrich SCFA-producing bacteria via trophic ecological competition. (2) Butyrate may act as a core effector molecule mediating tight junction–mucus layer barrier repair through dual pathways (HDAC inhibition and GPR receptor activation). (3) Polysaccharides may induce M2 macrophage polarization and inhibit the IL-6/STAT3 pathway to rebalance Th17/Treg subsets, completing hierarchical immune microenvironment remodeling. In contrast to saponins whose dual metabolic regulation (SCFA + bile acid) occurs mainly at the midstream of the axis, polysaccharides tend to support a positive feedback loop across the entire axis by supplying upstream substrates. Accordingly, in intervention strategies targeting the microbiota–metabolism–immunity axis in CAC, polysaccharides may be considered a valuable complementary component, as shown in Figure 4. Representative polysaccharide-related studies, including their sources, disease models, microbiota/metabolite changes, immune regulation, and barrier-protective effects, are summarized in Supplementary Table S4 [81,82,85,87,88,89,90,91,92,93,94].

## 3. Modulating Gut Microbiota Composition

### 3.1. The Crosstalk Between Gut Microbiota and CAC

Gut microbiota, a diverse community of bacteria, viruses, fungi and protozoa [95], has been suggested to be closely linked to host metabolism, immune function and intestinal barrier integrity. Accumulating evidence suggests that gut dysbiosis may act not merely as a concomitant feature of IBD, but potentially as a contributor to CAC initiation and progression [7].

Mechanistically, gut microbiota has been proposed to mediate the inflammation-to-cancer transition via three core pathways: (1) Pathogens may secrete genotoxins (e.g., colibactin) to induce oncogenic mutations [96] and pro-inflammatory mediators (e.g., LPS) that activate TLR4-dependent cascades, triggering NF-κB/STAT3 pathways and potentially sustaining a tumor-promoting microenvironment [17,97]. (2) Pathogenic taxa may disrupt the mucosal barrier, increase intestinal permeability, and drive inflammation-fueled tumorigenesis [83]. (3) Microbiota-derived SCFAs—critical energy sources for colonic epithelial cells—are thought to maintain barrier function and immune homeostasis [84,98]; reduced SCFA production in dysbiosis may impair epithelial repair and potentially accelerate inflammation-cancer transformation [9]. Thus, remodeling gut microbiota and elevating SCFAs to reinforce barrier function and suppress inflammation have been proposed as a core strategy against CAC.

### 3.2. Dysbiosis Signatures in CAC

CAC-associated gut dysbiosis has been reported to exhibit a characteristic signature: enrichment of pro-inflammatory/pro-tumorigenic bacteria and depletion of protective commensals.

*F. nucleatum*, considered the most well-characterized CAC-associated pathogen [99], is a Gram-negative anaerobe primarily residing in the oral cavity. Its colonic colonization has been correlated with IBD incidence and tumor invasiveness [100], with higher abundance in CAC tissues/feces than non-cancer IBD patients and detection rate reportedly increasing with tumor stage [101,102]. Mechanistically, its adhesin FadA has been shown to bind E-cadherin to activate the β-catenin pathway, potentially promoting epithelial hyperproliferation and epithelial–mesenchymal transition (EMT) [102]; it may also recruit tumor-associated macrophages (TAMs) and suppress the anti-tumor cytotoxicity of natural killer (NK) cells and T lymphocytes, potentially impairing immune surveillance [11,99].

Concurrently, protective commensals (e.g., *Bifidobacterium*, *Lactobacillus*, butyrate-producing *Faecalibacterium prausnitzii* and *Roseburia*) have been reported to be depleted in CAC [103,104], potentially leading to reduced colonic SCFAs (especially butyrate, a key energy source for intestinal epithelial cells). Butyrate deficiency has been suggested to impair barrier integrity, facilitating LPS translocation and chronic inflammation. Meanwhile, butyrate deficiency may inactivate GPR43, downregulate anti-inflammatory cytokines (e.g., TGF-β), and disrupt immune tolerance [105]. Reduced *Akkermansia muciniphila* (a mucus homeostasis regulator) has been correlated with mucosal damage and exacerbated inflammation [20], further compounding the loss of colonic SCFAs.

### 3.3. Two Pathways by Which TCM Active Components Modulate Gut Microbiota

TCM bioactive ingredients have been proposed to modulate the gut microbiota via two complementary, potentially synergistic pathways.

(1) Direct inhibition of pathogenic/opportunistic pathogens

Certain alkaloids, phenolic acids, and polyphenols have been reported to exert direct bacteriostatic effects or suppress microbial virulence factors, constituting a potential “source control” strategy against pro-tumorigenic microbiota. Studies have demonstrated that berberine significantly inhibits *F. nucleatum* growth and adhesion in vitro [106], and suppresses LPS release from diverse Gram-negative pathogens [107]. This direct inhibition is hypothesized to reduce pro-inflammatory signal input and alleviate aberrant mucosal immune activation.

(2) “Prebiotic-like” ecological niche reconstruction

Flavonoids, polyphenols, and polysaccharides exhibit low upper-gastrointestinal absorption, with most prototypes reaching the colon for microbial metabolism or fermentation [108]. These compounds conform to the classical definition of prebiotics [109]: they may serve as fermentable carbon sources for *Bifidobacterium*, *Lactobacillus* and other probiotics, and have been reported to stimulate the proliferation and metabolic activity of butyrate producers (e.g., *Faecalibacterium*, *Roseburia*) [110]. This effect is thought to restore SCFA-producing commensal abundance, potentially laying the microecological foundation for subsequent metabolite elevation and intestinal barrier repair [111].

### 3.4. Representative Evidence

Berberine has shown chemopreventive potential against CAC in preclinical models. In AOM/DSS-induced CAC mice, prophylactic berberine administration markedly reduces tumor burden and inflammatory scores, accompanied by favorable microbiota shifts: elevated abundance of *Lactobacillus rhamnosus* and suppressed levels of pro-inflammatory taxa such as *Proteobacteria* [45]. Further studies suggest that berberine may counteract *F. nucleatum*-mediated tumor promotion, primarily via microbiota remodeling and tumor immune microenvironment regulation [106].

Curcumin has also shown anti-CAC efficacy in preclinical studies. In IL-10 knockout mice (a spontaneous colitis-associated CRC model), curcumin intervention alleviates colitis symptoms and reduces tumor incidence, concurrent with restored *Lactobacillus* and *Bifidobacterium* abundances and decreased *Enterobacteriaceae* levels [112]. Recent AOM/DSS model studies verify that curcumin fosters the proliferation of *Butyricicoccus* and other butyrate producers, elevating fecal butyrate levels and further supporting its microbiota–metabolism-mediated anti-carcinogenic effects [113].

Targeting *Akkermansia muciniphila* represents a promising TCM-related intervention strategy. Animal studies demonstrated that supplementation with viable *Akkermansia muciniphila* or its major outer membrane protein Amuc 1100 ameliorates DSS-induced colitis and impedes CAC progression, via reinforcing the mucus layer, upregulating tight junction proteins (ZO-1, Occludin) and modulating CD8^+^ T cell activity [20]. These findings highlight *Akkermansia muciniphila* enrichment as a potential key target for microbiota-directed TCM interventions.

**Figure 5:**
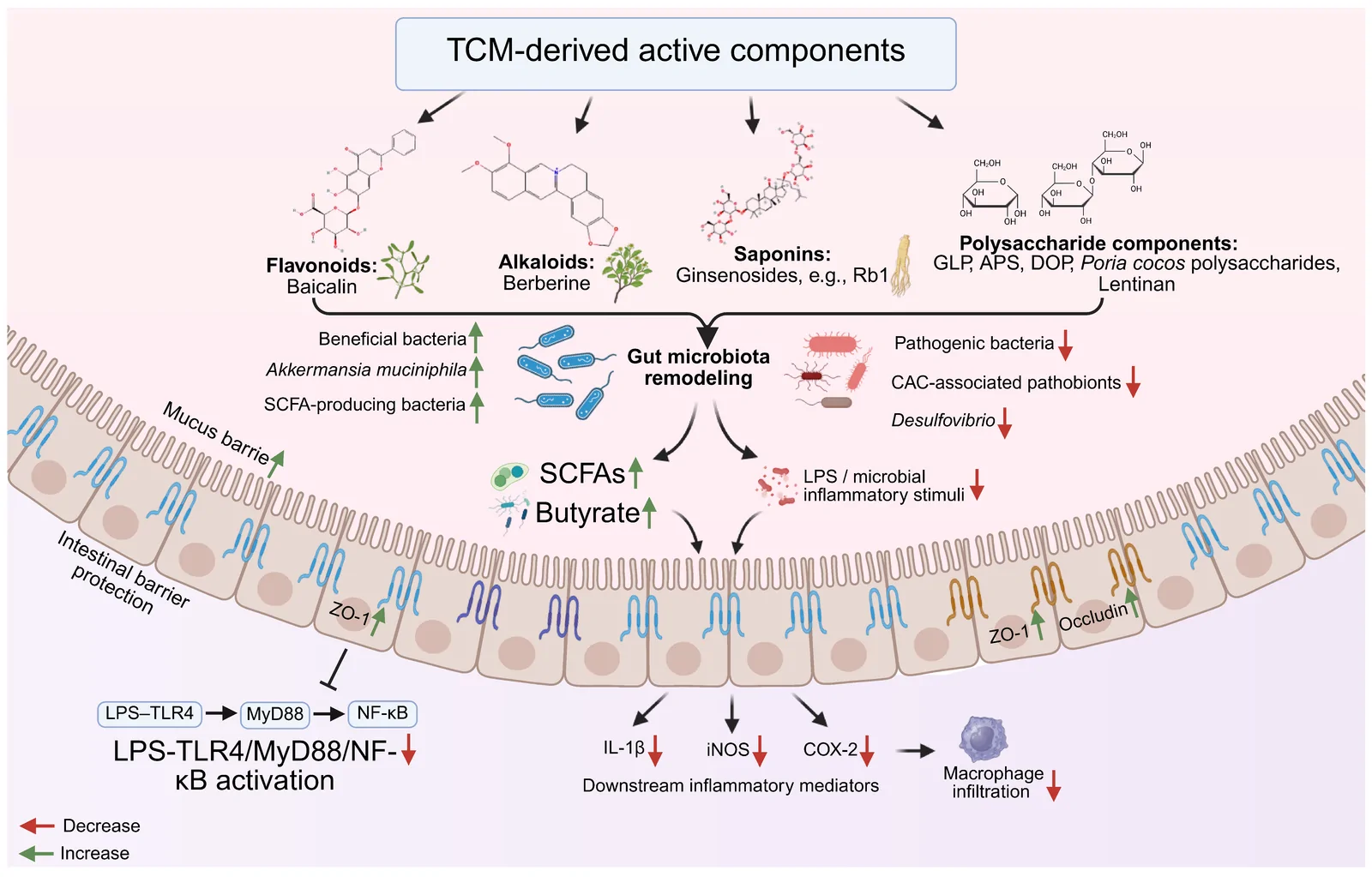
Gut microbiota remodeling by TCM-derived active components. TCM-derived flavonoids, alkaloids, saponins, and polysaccharides can adjust the composition of gut bacteria, increase beneficial bacteria and those that produce SCFAs, and to some extent reduce harmful bacteria and potential pathogens related to colorectal cancer.

TCM active ingredients (e.g., berberine, curcumin) and probiotic mediators (e.g., *A. muciniphila*) have been proposed to reshape the gut microbiota ecological niche via a dual pathway: suppressing pro-inflammatory/pro-tumorigenic taxa and enriching beneficial commensals/SCFA producers. This microecological remodeling is thought to directly modulate the synthesis and release of key metabolites (notably SCFAs), thereby potentially driving intestinal barrier repair and immune rebalancing to block CAC development and progression, as shown in Figure 5.

## 4. Regulating Microbial Metabolites

### 4.1. Microbial Metabolites as Key Mediators in the Microbiota–Host Crosstalk

Gut microbial metabolites are considered pivotal messengers mediating host–microbe interactions, produced by microbiota metabolizing dietary components, host secretions and exogenous substances [12,114]. They act on intestinal epithelial/immune cells and distal organs, potentially shaping host physiology and pathology [15]. In CAC, dysregulated microbial metabolites have been proposed to drive the transition from gut dysbiosis to inflammation-driven carcinogenesis [9]. The most studied microbial metabolites are SCFAs, bile acids and tryptophan metabolites, with SCFAs being the primary target of TCM active ingredients due to their roles in intestinal homeostasis, immune regulation and anti-tumor effects [23,115].

### 4.2. Short-Chain Fatty Acids: The Central Metabolic Axis

SCFAs, main end-products of microbial fermentation of dietary fiber and resistant starch (acetate, propionate, butyrate; molar ratio of ~60:20:20) [116], are synthesized by *Faecalibacterium prausnitzii*, *Roseburia*, *Eubacterium rectale* and Clostridium clusters IV/XIVa [117]. These butyrate-producing bacteria have been reported to be significantly depleted in CAC, potentially leading to insufficient SCFA synthesis [118].

### 4.3. The Core Mechanism of Action of Butyric Acid

Butyrate, the primary energy source for colonic epithelial cells (~70% energy supply) [116], is proposed to exert protective effects via two core pathways:

(1) As a ligand for GPR43 (FFAR2) and GPR109A (HCAR2) on epithelial/immune cells [13], butyrate has been shown to inhibit NF-κB signaling to reduce pro-inflammatory cytokines (IL-6, TNF-α, IL-1β) [13,119] and induce Treg differentiation/IL-10 secretion to sustain immune tolerance [120,121].
(2) As an HDAC inhibitor, butyrate has been suggested to epigenetically regulate gene expression: (1) upregulating tight junction proteins (ZO-1, Occludin, Claudin-1) for barrier repair [122]; (2) blocking NF-κB/STAT3 signaling to abrogate pro-inflammatory/pro-carcinogenic effects [123]; and (3) inducing tumor cell cycle arrest and apoptosis [124].

### 4.4. TCM-Derived Active Components Promote SCFA Production

TCM-derived active components have been proposed to enhance SCFA biosynthesis via two complementary mechanisms: (1) serving as direct fermentable substrates for intestinal microbiota and (2) indirectly enriching SCFA-producing microbial communities by remodeling gut microbiota composition.

Quercetin, a dietary flavonoid abundant in fruits and vegetables, exhibits poor intestinal absorption after oral administration, with most unabsorbed molecules reaching the colon for microbial metabolism [125]. In DSS-induced colitis mice, quercetin intervention drastically reshapes gut microbiota structure, elevating the abundance of beneficial taxa including *Bacteroides*, *Bifidobacterium* and Clostridium cluster XIVa, and increasing fecal SCFA levels—particularly butyrate and propionate [126]. Mechanistic studies suggest that unabsorbed quercetin acts as a fermentation substrate, and its metabolites exert prebiotic-like effects; quercetin also modulates the AhR pathway and inhibits pro-inflammatory cytokine release, indirectly facilitating SCFA synthesis and exerting anti-inflammatory effects [28].

Resveratrol, a polyphenol with extremely low oral bioavailability [127], has been reported to have over 70% reaching the colon intact [128]. In AOM/DSS-induced CAC mice, it reduces tumor incidence, enriches *Lactobacillus*/*Bifidobacterium*, suppresses opportunistic pathogens, and elevates fecal butyrate (positively correlated with butyrate-producing *Lachnospiraceae*) [129,130,131].

APS, a major active component of *Astragalus membranaceus* with high-molecular-weight sugar chains, resists upper gastrointestinal hydrolysis and reaches the colon as a fermentable substrate [132,133]. In DSS-induced colitis mice, it enriches probiotics (*Bifidobacterium*, *Lactobacillus*) and butyrate-producing *Faecalibacterium*, significantly increasing cecal SCFAs (most notably butyrate) [81,134,135]. APS-derived SCFAs are proposed to exert anti-inflammatory/barrier-protective effects via GPR43 activation and NF-κB inhibition [120,121].

In addition to SCFAs, bile acid and tryptophan metabolites are also considered critical mediators of the microbiota–host crosstalk, although research on their regulation by TCM ingredients in CAC prevention and treatment remains relatively limited.

Bile acid metabolism: Gut microbiota has been proposed to mediate the conversion of primary bile acids synthesized by the liver (cholic acid, chenodeoxycholic acid) into secondary bile acids (deoxycholic acid, lithocholic acid). These secondary bile acids have been suggested to regulate intestinal inflammation and epithelial cell proliferation by activating FXR and TGR5 [136]. Gut dysbiosis may impair microbial bile acid deconjugation and dehydroxylation, reducing secondary bile acid production, downregulating the FXR-FGF15 axis and disrupting TGR5 expression—all of which have been hypothesized to promote CAC pathogenesis [137]. Emerging evidence suggests that certain TCM-derived active components modulate the microbiota–bile acid axis to exert biological effects. For instance, ginsenoside (Compound K, Rk3) has been reported to enrich beneficial microbes (*Bacteroides*, *Akkermansia*) and inhibit pathogens such as *Clostridium scindens*, thereby altering secondary bile acid synthesis and composition, activating FXR/TGR5 signaling to exert anti-inflammatory and anti-carcinogenic effects [69]. Berberine has been reported to inhibit gut microbial 7α-dehydroxylase activity, reduce the production of secondary bile acids (DCAs), activate the FXR pathway, regulate cholesterol metabolism and alleviate intestinal inflammation [138]. While preliminary studies support TCM-mediated regulation of microbial bile acid metabolism, the precise mechanisms underlying their potential anti-CAC effects require further elucidation.

Tryptophan metabolites: Gut microbiota metabolizes dietary tryptophan into indole and its derivatives (indole-3-acetic acid, indole-3-propionic acid), which act as endogenous ligands for AhR. AhR activation has been hypothesized to enhance intestinal barrier function and modulate mucosal immunity [139]. Existing studies indicate that some flavonoids indirectly regulate the tryptophan metabolic pathway and AhR signaling by reshaping gut microbiota composition or activity [140]. However, direct evidence for flavonoid-mediated regulation of tryptophan metabolism remains scarce, and the exact intestinal protective mechanisms require further experimental validation.

**Figure 6:**
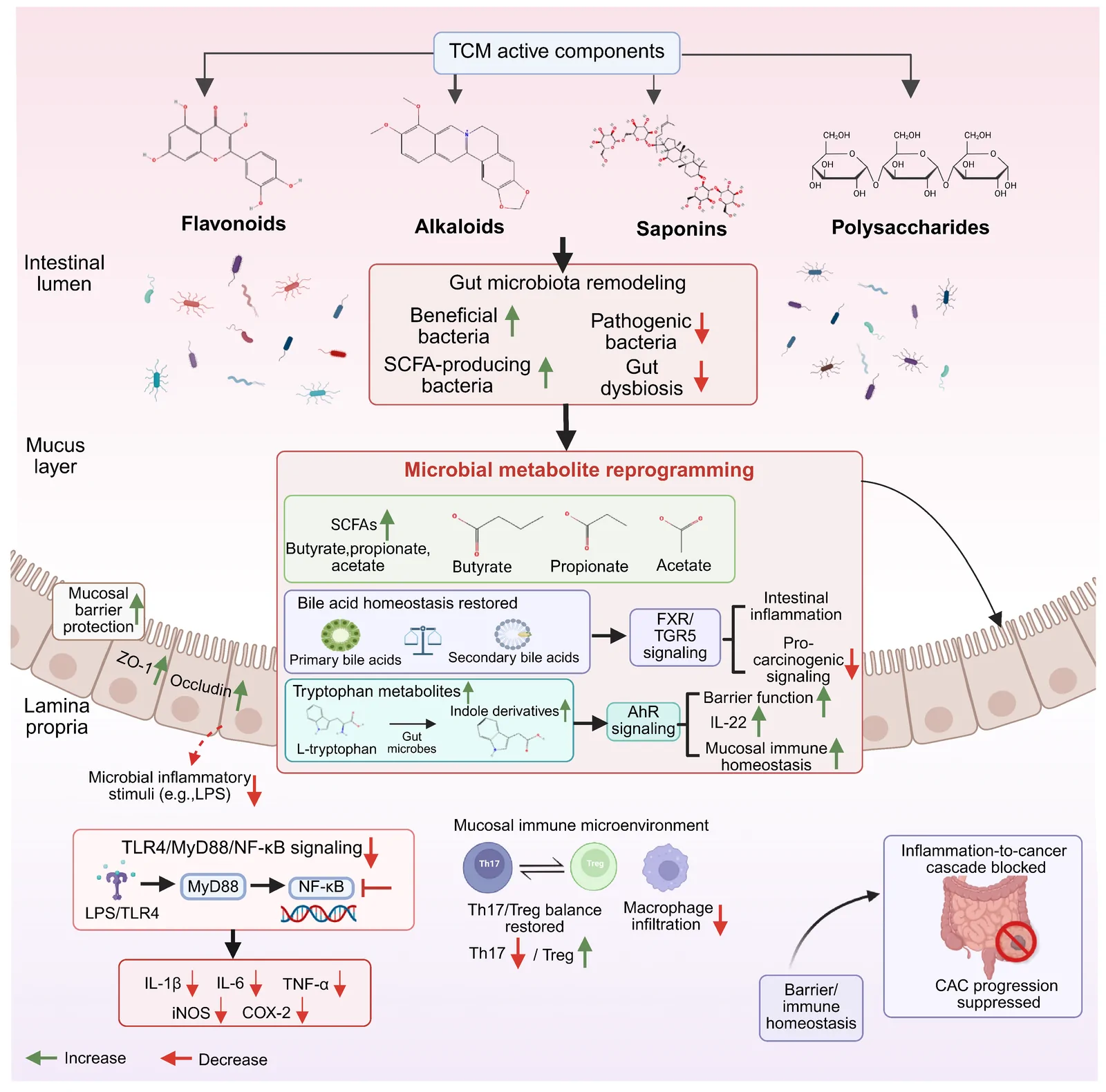
Microbial metabolite reprogramming by TCM-derived active components. It may facilitate microbial metabolite reprogramming, partly through elevated SCFAs, bile acid homeostasis restoration, and enhanced tryptophan-derived AhR signaling, potentially improving barrier integrity and impacting pro-carcinogenic inflammation.

TCM-derived active components have been proposed to boost butyrate synthesis via two core pathways: acting as fermentable substrates for gut microbiota, and enriching SCFA-producing microbial communities. Butyrate is proposed to exert multi-faceted protective effects by activating GPR43/GPR109A signaling and inhibiting HDAC activity, including blocking NF-κB/STAT3 signaling, inducing Treg differentiation and preserving intestinal barrier integrity. These metabolic modifications are further hypothesized to remodel the intestinal immune microenvironment, regulating key processes such as Th17/Treg balance, macrophage polarization and inflammasome activation to potentially block CAC progression, as depicted in Figure 6.

## 5. Regulating Immune Homeostasis: A Core Strategy for Blocking Inflammation-Driven CAC

The transition from chronic colitis to CAC has been closely associated with sustained inflammation-mediated mechanisms, in which a dysregulated immune microenvironment is considered to act as both an initiator and a sustainer of tumorigenesis [141,142]. Within this pathological context, persistent activation of innate and adaptive immune responses may establish a feedback loop in which pro-inflammatory cytokines promote intestinal epithelial cell proliferation and survival while attenuating anti-tumor immune surveillance, thereby fostering a microenvironment permissive to tumor progression [143]. Accumulating preclinical evidence, predominantly from rodent models of chemically induced colitis-associated tumorigenesis (e.g., AOM/DSS) and from in vitro systems, suggests that diverse bioactive compounds derived from TCM may modulate this dysregulated immune response by targeting cytokine networks, influencing immune cell polarization, and regulating aberrant inflammasome activation. However, these models do not fully recapitulate the pathophysiology of human ulcerative colitis-associated neoplasia, and the following mechanistic observations should therefore be interpreted as hypothesis-generating rather than as clinically established effects.

### 5.1. Attenuation of Pro-Inflammatory Cytokine Network

IL-6, TNF-α, and IL-1β are the three core pro-inflammatory cytokines that form the inflammatory engine potentially fueling CAC progression. IL-6 activates the JAK/STAT3 signaling cascade in intestinal epithelial cells, thereby potentially enhancing cell proliferation, survival, and angiogenesis while inhibiting apoptosis [144,145]. TNF-α drives NF-κB-dependent transcriptional upregulation of chemokines and adhesion molecules, which may facilitate continuous immune cell infiltration into the colonic mucosa and sustain chronic inflammation [146]. IL-1β, primarily processed and matured via inflammasome activation, further amplifies the inflammatory microenvironment and may promote EMT [147].

Multiple TCM-derived active components have exhibited potent inhibitory effects on these pro-inflammatory cytokines in the AOM/DSS-induced CAC model. For example, berberine, a representative isoquinoline alkaloid from *Coptis chinensis*, has been reported to significantly decrease colon tissue levels of IL-6, TNF-α, and IL-1β in this model. Mechanistically, this effect is presumably mediated by inhibiting IκB-α phosphorylation, which stabilizes IκB-α and prevents its proteasomal degradation, thereby blocking nuclear translocation of the NF-κB p65 subunit [148]. Similarly, curcumin has been reported to suppress the AKT/NF-κB signaling pathway in both CAC induction and progression. It also reduces IL-6 activation while downregulating phosphorylated STAT3 and c-MYC expression in colon tissue, potentially disrupting the IL-6/STAT3 positive feedback loop that sustains the pro-tumor inflammatory microenvironment [149]. These mechanisms were observed in preclinical models and still need to be further validated in clinical settings.

### 5.2. Rebalancing Adaptive Immunity and Innate Immune Cell Polarization

Beyond soluble inflammatory mediators, the composition of immune infiltrates is thought to substantially shape CAC outcomes, with the Th17/Treg axis frequently proposed as a key regulatory node. In preclinical CAC models, IL-17A-producing Th17 cells undergo marked expansion [150], together with a relative depletion of Foxp3^+^ Treg cells [151]. This imbalance has been suggested to contribute to chronic mucosal inflammation and impaired immune surveillance. Additionally, in late-stage CAC, TAMs have been observed to shift from pro-inflammatory M1 macrophages toward an immunosuppressive M2 macrophages. M2-polarized macrophages are associated with secretion of immunosuppressive mediators (e.g., IL-10, TGF-β) and matrix metalloproteinases, which may facilitate tumor invasion and metastasis. Recent studies have further proposed that TAMs contribute to remodeling of the tumor collagen matrix, offering additional perspectives on their potential pro-tumorigenic roles in colorectal cancer [152].

TCM-derived active components have been reported to influence immune cell polarization in preclinical settings. Baicalin has been associated with attenuation of Th17/Treg imbalance in the colonic lamina propria of AOM/DSS mice [153], with apparent reductions in RORγt expression and increases in Foxp3^+^ cell frequency [154], effects that have been tentatively linked to inhibition of IL-6/STAT3 signaling pathway [155,156]. Astragaloside IV, a cycloartane-type saponin from *Astragalus membranaceus*, has been reported to be associated with a shift from M2 toward M1 macrophage polarization in the AOM/DSS tumor microenvironment, characterized by changes in iNOS/CD86 and Arg-1/CD206 expression, with suggested involvement of STAT3 and NF-κB signaling [95,157].

Nevertheless, the majority of these observations are derived from single-model preclinical studies with heterogeneous dosing regimens and limited mechanistic resolution in CAC-specific contexts. Direct evidence linking these immune-modulatory observations to reduced tumor incidence or improved outcomes in patients with inflammatory bowel disease remains absent.

### 5.3. Regulation of NLRP3 Inflammasome Activity: A Context-Dependent Mechanism

The NLRP3 inflammasome occupies a dual and context-dependent position in CAC pathogenesis. While excessive NLRP3 activation drives IL-1β and IL-18 maturation and may sustain chronic mucosal inflammation, experimental evidence has paradoxically indicated that NLRP3 may also function as a negative regulator of tumorigenesis during colitis-associated carcinogenesis, likely by supporting epithelial barrier integrity and IL-18-mediated anti-tumor responses [158,159]. Accordingly, the relationship between NLRP3 activity and CAC outcomes appears to be non-linear and context-dependent, suggesting that therapeutic strategies aiming for precise calibration, rather than wholesale inhibition, of NLRP3 activity may be more biologically rational.

Inflammatory activation induces NF-κB-driven transcription of NLRP3 and pro-IL-1β, which can lead to excessive inflammasome activation. Curcumin has been reported to mitigate acute inflammatory injury by inhibiting NLRP3 inflammasome activation through multiple mechanisms, including blocking K^+^ efflux, stabilizing lysosomes, and scavenging reactive oxygen species (ROS) [160]. However, complete suppression of NLRP3 signaling may not be desirable in CAC, because NLRP3- and caspase-1-dependent IL-18 signaling has been implicated in maintaining epithelial barrier integrity and supporting anti-tumor immune surveillance in experimental colitis-associated tumorigenesis [159,161]. Therefore, current evidence suggests that TCM-derived active components should be more appropriately considered as potential modulators of aberrant inflammasome activity rather than simple NLRP3 inhibitors. This concept requires further validation in CAC-specific models with cell-type disease, stage, and resolved analyses.

### 5.4. Convergence of Key Signaling Pathways: A Potential Multi-Target Feature of TCM-Derived Active Components

**Figure 7:**
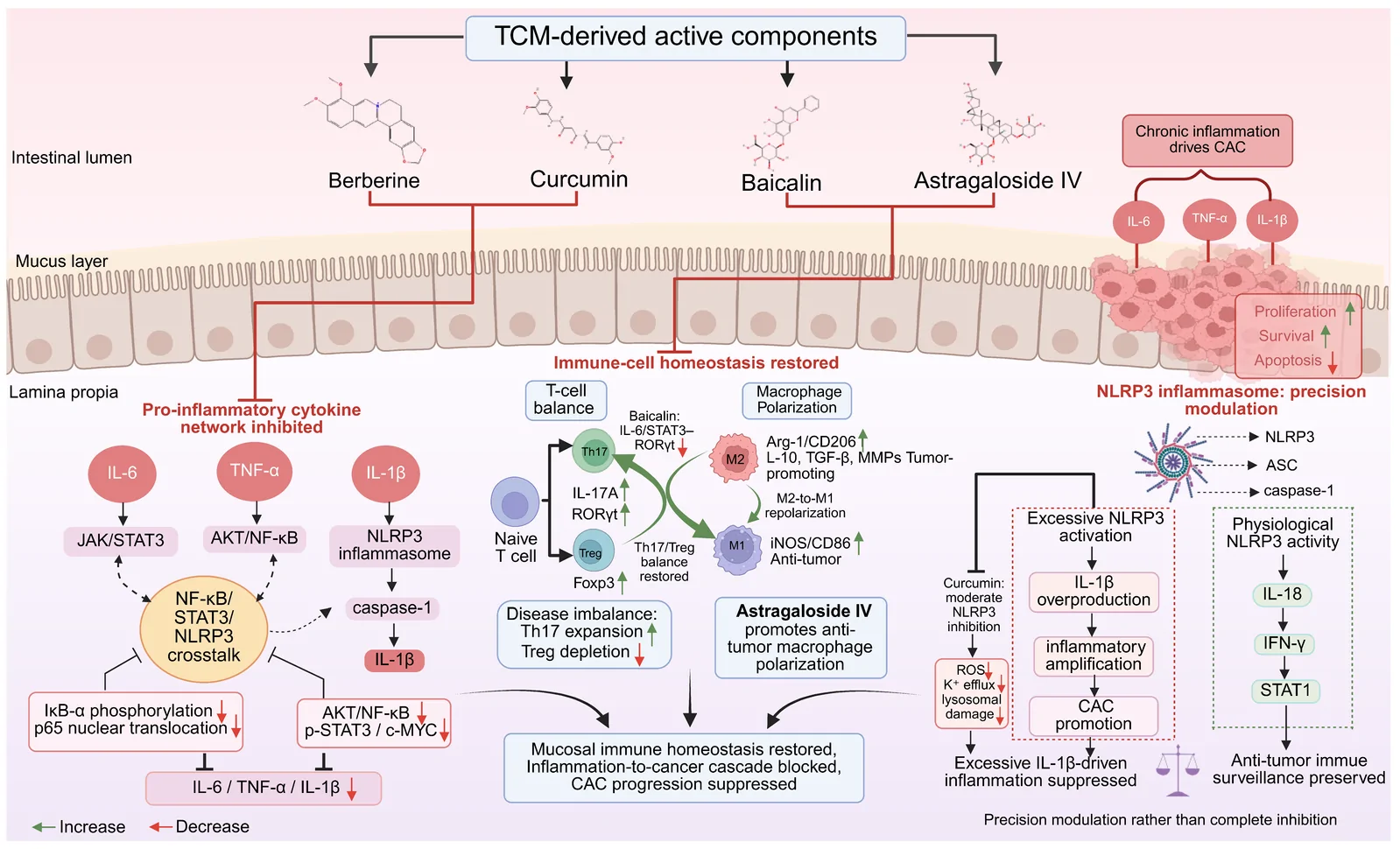
Immune microenvironment modulation by TCM-derived active components. TCM active components are associated with reduced pro-inflammatory cytokine signaling, improved T-cell and macrophage balance, and regulation of NLRP3 inflammasome activity, with potential relevance to limiting CAC-related progression.

NF-κB, JAK/STAT3, and NLRP3 inflammasome signaling is closely interconnected in the CAC inflammatory microenvironment rather than acting as independent pathways. NF-κB activation may promote the transcription of IL-6, NLRP3 and pro-IL-1β, thereby contributing to STAT3 activation and inflammasome priming; meanwhile, persistent STAT3 signaling can further sustain inflammation-related survival and proliferation signals, forming a potential feed-forward inflammatory circuit [162,163,164]. Within this network, some TCM-derived active components have been reported to modulate multiple inflammatory signaling nodes in preclinical models, for example, berberine (targeting NF-κB, NLRP3, and STAT3) [21,165] and curcumin (targeting NF-κB and STAT3) [166,167]. These findings suggest that the multi-target characteristics of TCM-derived active components may be relevant to the regulation of inflammation-driven CAC progression as depicted in Figure 7.

However, most available evidence remains preclinical, and the potential advantages of multi-pathway modulation should not be directly interpreted as confirmed clinical efficacy. Further studies using standardized CAC models, cell-specific mechanistic analyses, and clinically relevant validation are needed to clarify whether coordinated regulation of these signaling networks can contribute to CAC prevention or intervention.

## 6. Discussion

The studies reviewed here point to a common theme: TCM-derived active components can affect several processes implicated in CAC, including gut microbial ecology, microbial metabolism, epithelial barrier function, and mucosal immunity. This interpretation must remain cautious. Most evidence comes from AOM/DSS models, experimental colitis induced by DSS or TNBS, sporadic colorectal cancer models, or cell-based experiments. These studies are informative for mechanisms, but they do not demonstrate clinical efficacy in patients with CAC [19,79].

### 6.1. Class-Specific Mechanistic Patterns

Comparison across chemical classes reveals considerable overlap as well as some useful differences. The boundaries are not absolute, because the activity of an individual compound depends on its dose, formulation, metabolism, and the model in which it is tested.

Among the flavonoids, baicalin, luteolin, quercetin, and wogonin have mainly been studied for their effects on inflammatory and oxidative stress pathways. Reported actions include inhibition of NF-κB, STAT3, MAPK, and NLRP3 signaling, lower expression of pro-inflammatory mediators, and improvement in epithelial barrier markers. Curcumin, a non-flavonoid polyphenol, shows a partly overlapping profile. Changes in microbial composition or metabolite production have also been reported for several of these compounds. Their low and variable oral bioavailability, however, makes it difficult to relate systemic effects directly to the administered parent compound [25].

Berberine is the most extensively studied alkaloid in this context. Its reported effects span microbial community structure, SCFA and bile acid metabolism, barrier function, and NF-κB- and IL-6/STAT3-related inflammation. The high intestinal exposure of berberine relative to its systemic availability suggests that local actions and microbial biotransformation may be especially important. Whether the same mechanisms operate in human CAC has not yet been shown.

Ginsenosides and astragaloside IV have been linked mainly to immune regulation, particularly macrophage polarization, cytokine production, and remodeling of the inflammatory tumor microenvironment. Much of this work was performed in sporadic colorectal cancer or other non-CAC models, so its relevance to CAC is indirect.

Polysaccharides from Ganoderma lucidum and Astragalus are more often discussed in relation to the gut microbiota. In experimental colitis and CAC-related models, they have been associated with enrichment of potentially beneficial bacteria, increased SCFA production, improved barrier integrity, and changes in the Th17/Treg balance [81,82]. Interpretation across studies is difficult because polysaccharide preparations vary markedly in source, extraction and purification, molecular-weight distribution, monosaccharide composition, and linkage structure.

Thus, the four groups converge on a limited set of biological processes but do not necessarily act through a single shared pathway. The microbiota–metabolism–immunity axis is best viewed as an organizing framework for these observations, not as proof that every reported anti-tumor effect is microbiota-dependent or applicable to human CAC.

### 6.2. Limitations of the Current Evidence Base

The main constraint is the limited disease specificity of the available models. AOM/DSS reproduces important features of inflammation-associated tumorigenesis, but not the prolonged course, treatment history, host diversity, or molecular heterogeneity of human CAC. DSS and TNBS models primarily represent inflammatory injury, while sporadic colorectal cancer models and isolated cell systems offer only indirect support.

Methods also differ substantially between studies. Animal strain, sex, age, housing, induction protocol, dose, route, treatment schedule, and outcome definition are rarely consistent. The problem is greater for extracts and polysaccharides, for which botanical origin, processing, chemical composition, purity, and structural characterization are often incompletely reported. These differences limit reproducibility and make dose or exposure comparisons unreliable.

Most experiments also emphasize short-term changes in cytokines, signaling proteins, microbiota profiles, or tumor burden. Far less is known about pharmacokinetics, intestinal exposure, active metabolites, target engagement, dose–response relationships, long-term toxicity, or recurrence. On this basis, the evidence remains exploratory.

### 6.3. Translational Research Priorities

The first priority is to connect chemical identity and exposure with biological activity. Studies should use well-characterized, batch-consistent materials and report both intestinal and systemic exposure. Identification of active metabolites, together with dose–response and safety data, is particularly important for compounds with poor oral bioavailability and for chemically complex extracts or polysaccharides.

The causal role of microbiota also needs to be tested rather than inferred from parallel changes. Fecal microbiota transplantation, antibiotic perturbation, germ-free or gnotobiotic models, defined microbial consortia, and metabolite depletion or rescue can help determine whether a microbial or metabolic change mediates the treatment effect. Longitudinal sampling would further distinguish early treatment responses from changes that simply follow improvement in inflammation or tumor burden.

Multi-omics and single-cell or spatial approaches can help resolve interactions among microbial functions, metabolites, epithelial cells, and immune populations, but broad profiling alone will add little unless it is followed by targeted functional experiments. Non-coding RNAs may form part of this regulatory network; their contribution to the effects of TCM-derived active components requires direct experimental evidence [29].

Early clinical studies should use standardized interventions, define pharmacokinetic and pharmacodynamic endpoints, and monitor safety systematically. Diet, recent antibiotic exposure, concomitant medication, disease activity, and baseline microbiome composition should be recorded prospectively because each may influence the observed response. Given the long interval over which CAC develops, validated inflammatory, epithelial, microbial, or metabolic markers may serve as intermediate outcomes, but they cannot substitute for histopathological and clinical endpoints.

### 6.4. Limitations of the Present Review

This review provides a narrative, literature-based comparison of TCM-derived active components in CAC-related and inflammation-associated intestinal tumorigenesis models. Because the available studies differ substantially in model type, intervention form, dosage, route of administration, treatment duration, and outcome measures, the strength of evidence cannot be judged using a single uniform standard. In addition, some mechanistic evidence was drawn from experimental colitis, sporadic colorectal cancer, non-intestinal disease models, and cell-based systems. These studies help explain possible biological pathways but are less directly relevant to human CAC than dedicated CAC models. Therefore, the comparisons among flavonoids, alkaloids, saponins, and polysaccharides should be interpreted as a qualitative overview of shared and class-associated mechanisms, rather than as a hierarchy of efficacy.

## 7. Conclusions

TCM-derived flavonoids, alkaloids, saponins, and polysaccharides have been associated in CAC-related and inflammation-associated intestinal tumorigenesis models with changes in gut microbial composition, microbial metabolites, epithelial barrier function, inflammatory signaling, and mucosal immune responses. Viewed together, the available evidence supports the microbiota–metabolism–immunity axis as a useful framework for comparing these component classes at the mechanistic level. Flavonoids and alkaloids are more frequently reported in relation to inflammatory signaling and epithelial barrier protection, saponins in relation to microbiota-dependent metabolism and host–microbe interactions, and polysaccharides in relation to microbiota remodeling, SCFA production, and immune homeostasis. These patterns should be interpreted as mechanistic associations rather than evidence of clinical efficacy, because the current evidence remains heterogeneous, largely preclinical, and only partly supported by causal validation.

## Data Availability

No new data were created or analyzed in this study. Data sharing is not applicable to this article.

## Supplementary Materials

The following supporting information can be downloaded at: https://www.mdpi.com/article/10.3390/ijms27198836/s1.

## Abbreviations

The following abbreviations are used in this manuscript:

CAC	colitis-associated colorectal cancer
IBD	inflammatory bowel disease
UC	ulcerative colitis
CD	Crohn’s disease
CRC	colorectal cancer
TCM	traditional Chinese medicine
SCFAs	short-chain fatty acids
GPR43/GPR109A	G protein-coupled receptor 43/109A
HDAC	histone deacetylase
NF-κB	nuclear factor kappa-B
Treg	regulatory T cell
Th17	T helper 17 cell
LPS	lipopolysaccharide
TLR4	Toll-like receptor 4
JAK/STAT3	Janus kinase/signal transducer and activator of transcription 3
NLRP3	NOD-like receptor family pyrin domain-containing 3
FXR	farnesoid X receptor
TGR5	Takeda G protein-coupled receptor 5
AhR	aryl hydrocarbon receptor
F/B	firmicutes/bacteroidetes ratio
LRH	Lonicera rupicola Hook
TNBS	2,4,6-trinitrobenzene sulfonic acid
DSS	dextran sulfate sodium
AOM	azoxymethane
ZO-1	zonula occludens-1
IL-1β	Interleukin-1β
TNF-α	tumor necrosis factor-α
IL-6	Interleukin-6
SIgA	secretory immunoglobulin A
RORγt	retinoic acid receptor-related orphan receptor gamma t
PI3K/AKT	phosphatidylinositol 3-kinase/protein kinase B
TGF-β	transforming growth factor-β
ARG-1	arginase-1
HBG	Hordeum secalinum bioactive glycoside
TFGU	total flavonoids of Glycyrrhiza uralensis
CPT-11	irinotecan
ISL	isoliquiritigenin
ICA	Icariin
ACLY	ATP-citrate lyase
FAS	fatty acid synthase
HSL	hormone-sensitive lipase
ATGL	adipose triglyceride lipase
mTORC2	mechanistic target of rapamycin complex 2
ICAM-1	intercellular adhesion molecule-1
MCP-1	monocyte chemoattractant protein-1
COX-2	cyclooxygenase-2
MDA	malondialdehyde
GSH	glutathione
SOD	superoxide dismutase
BBR	berberine
DHBB	Dihydroberberine
EVO	Evodiamine
MyD88	myeloid differentiation primary response 88
OMT	Oxymatrine
DCs	dendritic cells
CPT	Camptothecin
HO-1/Nrf2	heme oxygenase-1/nuclear factor erythroid 2-related factor 2
AMP	Atractylodes macrocephala polysaccharide
PAMK	Atractylodes macrocephala Koidz
APS	Astragalus polysaccharide
GPS	Ginseng polysaccharide ameliorate
LBP	Lycium barbarum polysaccharide
ZOP-1	Zingiber officinale polysaccharide
HCPs	*Houttuynia cordata* polysaccharides
Gal-3	galectin-3
LAG3	lymphocyte activation gene-3
GLP	Ganoderma lucidum polysaccharide
MDSCs	myeloid-derived suppressor cells
GPP	Glycyrrhiza polysaccharide
MUC2	mucin 2; SCP, Schisandra chinensis polysaccharide
MPO	Myeloperoxidase
NO	nitric oxide
ROS	reactive oxygen species
DOP	Dendrobium officinale polysaccharide
EMT	epithelial–mesenchymal transition
TAMs	tumor-associated macrophages
NK	natural killer
FMT	fecal microbiota transplantation
16S rRNA	16S ribosomal RNA
