# Supplementary material for: Targeting the Microbiota–Metabolism–Immunity Axis: Active Components of Traditional Chinese Medicine in Colitis-Associated Colorectal Cancer
Source: Int J Mol Sci. 2026 Oct 3;27(19):8836. doi: 10.3390/ijms27198836 (PMC13665006; doi:10.3390/ijms27198836)
Supplement: Supplementary file 1 [file ijms-27-08836-s001.zip › ijms-4513986-supplementary.pdf]

Table S1. Summary of Flavonoids anti-CAC by targeting the microbiota-metabolism-immunity.

Chinese medicine	Active compounds	Models	Dose/concentration and route	Pharmacological effects	Mechanisms of action	Main findings	References
Kushen *Sophorae flavescentis radix*	Kurarinone and SFE	DSS-induced UC in C57BL/6 mice, RAW 264.7 cells.	1) In-vivo SFE: 100 mg/kg, 200 mg/kg. Kurarinone: 20 mg/kg, 40 mg/kg. 2) In-vitro Kurarinone, 1.875-30 μM; formal test at 2, 4, 8 μM, co-incubated with 1 μg/mL LPS for 24 h.	Anti-inflammatory, immune regulation, improve intestinal mucosal injury, and improve UC effects.	1) Regulate the balance and homeostasis between Th17/Treg cells, restore intestinal immune homeostasis, and improve colitis. 2) Downregulate the expression of JAK2 and STAT3 proteins in colon tissue, inhibit their phosphorylation activation, reduce the expression of downstream RORγt, and block the abnormal differentiation of Th17 cells. 3) Promote the secretion of SIgA in colon tissue, modulate the activity of RAW 264.7 cells to mediate and alleviate inflammatory responses.	1) Both SFE and its main active constituent kurarinone alleviated clinical symptoms and colon histopathological injuries in DSS-induced colitis mice in a dose-related manner. 2) Kurarinone exhibited prominent anti-inflammatory activity in activated RAW264.7 cells, and improved intestinal mucosal barrier-associated SIgA level in colitis model animals.	[1]
Yanshengrendong *Lonicera rupicola hook. f. & thomson*	LRH	DSS-induced UC in C57BL/6 mice.	In-vivo: DSS-induced UC model (2.5% DSS in drinking water for 7 days); intragastric administration. LRH flavonoids at 600, 800, 1000 mg/kg.	Antioxidant, anti-inflammatory, intestinal barrier protection, and regulation of intestinal microecology effects.	1) Regulate the expression of PI3CA and p-AKT proteins, and inhibit the overactivation of the PI3K/AKT signaling pathway. 2) Downregulate the transcription of IL-1β, IL-6, and TNF-α, and promote the expression of GF-β2 and ARG-1 in macrophages. 3) Upregulate the expression of tight junction protein (ZO-1) and repair the intestinal epithelial barrier. 4) Reshaping the microbial community structure to reduce inflammatory stimulation.	1) LRH flavonoids dose-dependently alleviated DSS-induced colitis, relieved weight loss, lowered DAI scores and restored colon length. 2) 16S rDNA sequencing showed LRH flavonoids reshaped disturbed gut microbiota composition and diversity in UC mice. 3) It mitigated intestinal oxidative stress, regulate M1/M2 macrophage polarization.	[2]
Damaicao *Hordeum secalinum*	HBG	AOM/DSS-induced CAC in C57BL/6N mice.	In-vivo: intragastric administration for 9 weeks. Hulless barley grass phenolic extracts (PE): 600 mg/kg/d; polysaccharide extracts (PSE): 400 mg/kg/d; lipid extracts (LE): 200 mg/kg/d.	Anti-colorectal cancer, anti-inflammatory, improve physiological condition, and microbiota regulation effects.	1) Regulate the Wnt/β-Catenin cascade reaction, interfering with the proliferation and transformation of cancer cells. 2) Inhibit the levels of pro-inflammatory cytokines mediated by the NF-κB signaling pathway, interrupting the inflammation-cancer transformation chain. 3) Multi-component synergistic regulation, jointly acting on multiple biological targets related to tumors.	1) PE significantly reduced tumor burden, relieved colon pathological injury and lowered pro-inflammatory mediators in AOM/DSS mice, showing effects comparable to 5-ASA. 2) PSE and LE partly decreased pro-inflammatory cytokines but failed to improve colon tumor-related indicators and gut microbiota obviously.	[3]

					4) Suppress protein over expression and maintain intestinal homeostasis.		
Gancao *Glycyrrhizae radix et rhizoma*	TFGU	CPT-11-induced UC in C57BL/6 mice.	In-vivo: CPT-11 (40 mg/kg) by intraperitoneal injection; TFGU(135 mg/kg) and ciprofloxacin were administered intragastrically for 10 days.	Correct fecal metabolic disorders, anti-inflammatory, intestinal mucosal barrier protection, and regulate intestinal flora effects.	1) Reshaping the gut microbiota structure disrupted by CPT-11 treatment.<br>2) Reverse CPT-11-induced fecal metabolic profile abnormalities, target regulation of purine metabolism.<br>3) Acts on amino acid metabolic pathways, improving the intestinal metabolic microenvironment.<br>4) Downregulate the protein expression of NLRP3 and Cleaved caspase-1 in tissues, reduce the release of IL-1β, block the cascade amplification of inflammatory signals, and alleviate colonic inflammatory responses.	1) Oral TFGU effectively relieved CPT-11-triggered colitis-related manifestations including weight loss, colon shortening and elevated DAI, and lowered colonic pro-inflammatory cytokine levels.<br>2) TFGU restored the disturbed gut microbiota composition and reversed fecal metabolic abnormalities in irinotecan-injured mice.	[4]
	ISL	AOM/DSS-induced CAC in BALB/c mice.	In-vivo: ISL at 30, 75, 150 mg/kg was intragastrically administrated six times weekly.	Anti-tumor, anti-inflammatory, microbiota homeostasis restoration, and antioxidant and antiviral effects.	1) Reduce pathogenic bacteria (Escherichia, Enterococcus), enrich beneficial symbiotic bacteria (Butyricicoccus), and remodel the gut microbiota structure.<br>2) By inhibiting the activation of NF-κB, block the inflammatory cascade reaction.<br>3) Target macrophage polarization to intervene in the tumor-associated inflammatory environment.<br>4) The metabolites produced by butyrate-producing bacteria synergize with ISL to strengthen the intestinal barrier and inhibit carcinogenesis.	1) ISL reduced tumor incidence and multiplicity, relieved inflammatory manifestations and lowered colonic pro-inflammatory mediator levels in AOM/DSS-treated mice in a dose-dependent manner.<br>2) ISL partially restored the disturbed gut bacterial community structure of CAC model mice, with high-dose ISL showing more obvious regulatory effects.	[5]

Huangqi *Astragali Radix*	Formononetin	RAW264.7cells, CT-26 cells, HCT116 cells, DSS-induced UC in C57BL/6 mice, AOM/DSS-induced CAC in BALB/c mice.	1) In-vivo: FN 25, 50, 100 mg/kg, intragastric gavage; prophylactic Formononetin 25, 50 mg/kg via gavage.<br>2) In-vitro: formononetin 1-4 μM for RAW264.7, 1.56-6.25 μM for colon cancer cell lines.	Anti-tumor proliferation, anti-inflammatory, antioxidant protection, regulate lipid metabolism and induce cell death effects.	1) Intervene in the mTORC2/Akt signaling axis, downregulate Rictor and mTOR protein expression, and inhibit Akt phosphorylation.<br>2) Target the lipid synthesis pathway, downregulate the expression of key lipogenic enzymes ACLY and FAS, and limit tumor growth.<br>3) Regulate lipid catabolism, adjust the expression of HSL and ATGL, and remodel the lipid microenvironment.<br>4) Coordinated Regulation of Autophagy and Apoptosis.<br>5) Block malignant transformation triggered by chronic inflammation and improve the intestinal immune microenvironment.	1) Formononetin relieved colitis-related manifestations and suppressed inflammatory cytokine release, and it inhibited the proliferation of CT-26 and HCT116 colon cancer cells in vitro.<br>2) In AOM/DSS-induced CAC mice, FN reduced tumor burden, improved intestinal mucosal injury, and restored the disturbed composition of gut microbiota; preventive administration yielded better outcomes than therapeutic intervention.	[6]
Yinyanghuo *Epimedii folium*	ICA	CT26-WT cells, HCT116 cells, RAW264.7 cells, THP-1 cells, AOM/DSS-induced CRC in BALB/c mice,	1) In-vivo: icariin 100 mg/kg, oral gavage.<br>2) In-vitro: icariin 10 μM, 20 μM for co-culture experiments.	Antitumor activity, immune regulation, Reshape the tumor microenvironment, and Enhance efficiency effects.	1) Targeting the PI3K/AKT signaling axis to block the key intracellular signaling that induces M2 polarization.<br>2) Prevent the transformation of macrophages M0 into M2 type, maintain or restore the anti-tumor microenvironment.<br>3) Block tumor-stroma communication and disrupt the microenvironment that favors tumor progression.<br>4) Improve the immunosuppressive state and activate the anti-tumor immune response in the tumor microenvironment.	1) Icariin exhibited negligible direct cytotoxicity against colorectal cancer cells, but it suppressed M2-macrophage-driven proliferation, migration and invasion of CRC cells under co-culture conditions.<br>2) In two in-vivo colorectal cancer mouse models, oral icariin reduced tumor burden, improved colonic pathological lesions and decreased M2-macrophage infiltration within tumor tissues.	[7]

Huangqin *Scutellariae radix*	Baicalin	TNBS-induced UC in SD mice, RAW264.7 cells.	1) In-vivo: baicalin 25, 50, 100 mg/kg, intragastric gavage; mesalazine 100 mg/kg as positive control. 2) In-vitro: baicalin at 5×10^{-4}, 1×10^{-4}, 5×10^{-5} M, stimulated with 1 μg/mL LPS.	Anti-inflammatory effect.	1) Downregulate the expression of TLR4 protein in colonic mucosa and RAW264.7 cells, and inhibit the upstream activation of the TLR4 pathway. 2) Reduce the levels of TNF-α, IL-1β, IL-6, and related proteins ICAM-1, MCP-1, and COX-2. 3) Inhibits the phosphorylation of downstream NF-κB p65 and IκB, blocking the transmission and activation of the TLR4/NF-κB pathway.	1) Baicalin reduced macroscopic and histological colon injury, lowered MPO activity and multiple pro-inflammatory mediators in TNBS-induced colitis rats in a dose-dependent manner. 2) Baicalin suppressed LPS-triggered inflammatory response and the over-production of inflammatory mediators in RAW264.7 macrophage cells.	[8]
		TNBS-induced UC in SD mice.	In-vivo: baicalin 25, 50, 100 mg/kg, intragastric gavage, once every two days for 14 days.	Anti-inflammatory and antioxidant and improve intestinal flora imbalance effects.	1) Regulate the Th17/Treg cell balance and exert an immunomodulatory effect. 2) Reduce MDA levels in colon tissue and increase the content of antioxidants GSH and SOD. 3) Reverse TNBS-induced gut microbiota dysbiosis, increase microbiota diversity, and reshape gut microbiota structure. 4) Restore and increase the levels of major short-chain fatty acids (especially butyrate) in the colon, and promote the production of beneficial metabolites.	1) Baicalin dose-dependently relieved colitis manifestations, improved colon histological damage, enhanced intestinal tight-junction-related protein expression and alleviated oxidative injury in TNBS-challenged rats. 2) Baicalin reshaped disturbed gut microbiota structure and elevated fecal short-chain fatty acid levels, especially butyrate, in UC model rats.	[9]

Table S2. Summary of Alkaloids anti-CAC by targeting the microbiota-metabolism-immunity.

Chinese medicine	Active compounds	Models	Dose/concentration and route	Pharmacological effects	Mechanisms of action	Main findings	References
Huanglian *Coptidis rhizoma*	BBR	AOM/DSS-induced CAC in C57BL/6 J mice.	In-vivo: berberine 50, 100 mg/kg, intragastric gavage once-daily.	Anti-tumor and Chemoprevention, maintain microecological balance, metabolic regulation, and anti-inflammatory and barrier protection effects.	1) Inhibit pathogenic bacteria (Desulfovibrio), promote the enrichment of SCFA-producing bacteria, and reshape microbiota balance.<br>2) Increase the levels of butyrate and other SCFAs, enhance metabolite-mediated protection, provide energy for the intestinal epithelium, and suppress a pro-inflammatory environment.<br>3) Upregulate the expression of ZO-1 and Occludin proteins to repair the colon mucosal barrier damaged by inflammation.<br>4) Downregulate the expression of TLR4, reduce the secretion of downstream IL-6, and inhibit p-STAT3, blocking the inflammation-cancer transformation signaling axis.	1) Berberine dose-dependently reduced tumor number and tumor load, inhibited colonic cell proliferation and alleviated intestinal barrier damage in CAC model mice.<br>2) Berberine reshaped the disturbed gut microbiota composition, elevated fecal short-chain fatty acid levels and decreased fecal LPS content in AOM/DSS-treated mice.	[10]
		AOM/DSS-induced CAC in C57BL/6 J mice.	In-vivo: berberine 100 mg/kg, intragastric gavage daily; two independent animal experiments with different intervention cycles.	Anti-tumor and Chemoprevention, anti-inflammatory, and intestinal microecology and metabolic regulation effects.	1) Regulate the structure of the gut microbiota, inhibit pro-inflammatory/pathogenic bacteria, and selectively enrich beneficial bacteria with protective effects.<br>2) Correct and reverse amino acid metabolic disorders that occur during CAC development, reduce harmful metabolic products, and restore metabolic homeostasis.<br>3) Promote SCFAs metabolism, provide energy for intestinal epithelial cells, and exert anti-inflammatory barrier protective effects.<br>4) Downregulate the expression of Ctnnb1 and Wnt genes in the Wnt signaling pathway, and block abnormally activated Wnt signaling.	1) Berberine alleviated colitis-related symptoms, improved intestinal mucosal barrier function, suppressed tumor growth, inhibited cell proliferation and promoted epithelial apoptosis in AOM/DSS-treated mice.<br>2) Berberine reshaped gut microbiota composition, elevated the abundance of multiple beneficial bacteria and increased fecal short-chain fatty acid contents in CAC model mice.	[11]

	AOM/DSS-induced CAC in C57BL/6 mice.	In-vivo: pre-administrated berberine 7.5, 15 mg/kg via intragastric gavage, 2-4 weeks before model establishment.	Preventive anti-tumor, anti-inflammatory, barrier enhancement, and, homeostatic regulation effects.	1) Downregulate the mRNA levels of TNF-α, IL-1β, IL-6, and COX-2, inhibit the activation of the JNK/STAT3 signaling pathway, and block inflammatory signal transmission.<br>2) Downregulate the expression of PCNA and Ki-67, blocking excessive proliferation of epithelial cells.<br>3) Upregulate the expression of intestinal tight junction proteins ZO-1 and Occludin, increase the number of goblet cells, and repair physical barrier defects.<br>4) Reshape the microbial community and maintain intestinal homeostasis.	1) Pre-administration of berberine alleviated colitis symptoms, reduced tumor number, improved colon pathological injury and enhanced intestinal barrier function in DSS- and AOM/DSS-challenged mice.<br>2) Berberine pretreatment restructured disturbed gut microbiota profile, raised the abundance of beneficial bacteria, and *Lactobacillus rhamnosus* supplementation further strengthened its preventive effect against colitis-associated carcinogenesis.	[12]
DHBB	DSS-induced UC in BALB/C mice.	In-vivo: dihydroberberine 12.5, 25, 50 mg/kg, berberine 50 mg/kg, azathioprine 13 mg/kg, intragastric administration once daily for 8 days.	Significant anti-colitis activity, mucosal tissue protection and anti-exudation, restoration of intestinal barrier function, and anti-inflammatory immune regulation effects.	1) Downregulate the protein expression of TLR4 and its downstream MyD88, targeting and inhibiting the TLR4/MyD88 recognition receptor axis.<br>2) Inhibit the phosphorylation of IκBα (p-IκBα), prevent its degradation, and block the activation and nuclear translocation of the NF-κB signaling pathway<br>3) Inhibit the cascade release of pro-inflammatory cytokines, reducing the synthesis and secretion of downstream pro-inflammatory cytokines.<br>4) Mediate a anti-inflammatory–barrier repair virtuous cycle to achieve structural and functional recovery of gut barrier function.	1) Dihydroberberine dose-dependently relieved colitis-related clinical symptoms and colon histopathological injury in DSS-challenged mice, and its therapeutic effect was superior to berberine and comparable to azathioprine.<br>2) Dihydroberberine elevated the expression of tight-junction proteins and mucins, and reduced the excessive release of multiple colonic pro-inflammatory mediators in UC model mice.	[13]

Wuzhuyu *Euodiae fructus*	EVO	DSS-induced UC in C57BL/6 mice, DSS-induced CRC in $(apc)^{Minc}$/Gpt c57Bl/6 Mice, SW480 cells.	1) In-vivo: evodiamine 10, 30 mg/kg, intragastric administration. UC mice were treated for 3 weeks, CRC mice for 8 weeks.<br>2) In-vitro: evodiamine 20-200 μM for cell viability assay, 100, 200 μM for subsequent cell functional tests.	Anti-inflammatory, anti-tumor formation, and cell protection and anti-proliferation effects.	1) Inhibit the activation of the NF-κB signaling pathway and block the cascade release of pro-inflammatory factors IL-6 and TNF-α.<br>2) Regulate S100A8/S100A9 protein expression, block inflammation-cancer transformation.<br>3) Inhibit the Wnt/β-catenin signaling pathway and suppress the transcription of downstream target genes (Cyclin D1, c-Myc).<br>4) Repair intestinal mucosal homeostasis and improve the tissue structure of the damaged intestine.	1) Evodiamine alleviated colitis-related symptoms and colon pathological damage in DSS-induced UC mice, and reduced tumor number and size in $(apc)^{Minc}$/Gpt colorectal cancer mice.<br>2) Evodiamine lowered multiple pro-inflammatory cytokine levels in colon tissues and serum, and inhibited the proliferation of SW480 human colon cancer cells in vitro.	[14]
		AOM/DSS-induced CAC in C57BL/6 mice.	In-vivo: evodiamine 40 mg/kg, intragastric gavage daily. Short-term colitis phase treated for 18 days; long-term tumor-bearing phase treated for 12 weeks.	Anti-inflammatory, mucosal barrier protection, and anti-tumor proliferation and pro-apoptotic effects.	1) Promote the enrichment of SCFA-producing probiotics, reduce the levels of pro-inflammatory pathogenic bacteria, and regulate the balance of gut microbiota.<br>2) Regulate metabolic pathways such as tryptophan and intervene in microbial metabolism.<br>3) Downregulate inflammation-cancer related signaling pathways such as Wnt, Hippo, IL-17, and NOD-like receptor signaling pathways.<br>4) Regulate host gene expression and inhibit intestinal inflammation and tumor formation.	1) Evodiamine relieved colitis-related manifestations, improved intestinal mucosal barrier injury and reduced tumor number and size in AOM/DSS-challenged mice.<br>2) Evodiamine suppressed colonic epithelial proliferation and promoted tumor-cell apoptosis, and reshaped the disturbed gut microbiota composition in CAC model mice.	[15]
Kushen *Sophorae flavescentis radix*	Matrine	DSS-induced UC in C57BL/6 mice.	In-vivo: matrine 10 mg/kg , 20 mg/kg , intragastric administration once daily for 7 days.	Anti-ulcerative colitis, intestinal barrier protection, microecological regulation, and anti-inflammatory effects.	1) Intervene in barrier dysfunction caused by intestinal inflammation and repair the damaged intestinal epithelial barrier.<br>2) Inhibit the PPAR-α signaling pathway, thereby suppressing the transcriptional activation of Slc27a1 and Fabp1, and reduce the release of pro-inflammatory factors (TNF-α, IL-6, IL-1β).<br>3) Reshape the structure of the gut microbiota and correct the loss of specific bacterial populations or the expansion of harmful bacteria caused by colitis.	1) Matrine alleviated clinical symptoms and colon histopathological injury, and reduced the over-production of multiple colonic pro-inflammatory cytokines in DSS-challenged mice.<br>2) Matrine elevated the expression of mucus-related and tight-junction proteins to restore intestinal mucosal barrier, and partially corrected gut microbiota dysbiosis in UC model mice.	[16]

	OMT	DSS-induced colitis in BALB/c mice.	In-vivo: oxymatrine 100 mg/kg, intragastric administration once daily for 10 days.	Significant anti-inflammatory activity, Potent immune modulation, and microecological balance restoration effects.	1) Downregulate the expression of Toll-like receptors (TLRs), block the phosphorylation and nuclear translocation of downstream NF-κB p65, and inhibit TLR/NF-κB signaling.<br>2) Reduce the proportion of inflammatory DCs in the intestinal mucosa, inhibit the expression of their co-stimulatory molecules (CD80, CD86), and regulate the function of inflammatory dendritic cells.<br>3) Reshape the intestinal microecological structure and improve the metabolic microenvironment to protect the intestines.<br>4) Form a regulatory axis of microbiota - TLR4-infDCs to synergistically improve intestinal inflammation.	1) Oxymatrine relieved clinical manifestations and colon histopathological injury, balanced pro-and anti-inflammatory cytokine levels in DSS-induced colitis mice.<br>2) Oxymatrine reduced the abundance of inflammatory dendritic cells and partly restored the disturbed gut microbiota composition in colitis model mice.	[17]
Xishu *Camptotheca acuminata* Decne	CPT	DSS-induced UC in C57BL/6 mice, RAW264.7 cells.	1) In-vivo: CPT 0.5, 1, 1.5 mg/kg, intragastric administration.<br>2) In-vitro: CPT 0.25, 0.5, 1 μmol/mL, pre-treatment before LPS stimulation.	Efficient anti-inflammation, mucosal and barrier repair, and inhibit oxidative stress kinase effects.	1) Inhibit LPS-induced phosphorylation of p65 protein, block the nuclear translocation of NF-κB and the activation of inflammatory genes.<br>2) Regulate AKT phosphorylation levels to intervene in the inflammatory activation signaling of cells.<br>3) Block the MAPK signaling pathway to reduce the cascade release of downstream pro-inflammatory mediators.<br>4) Downregulate the expression of key pro-inflammatory proteins iNOS and COX-2 in colonic tissue and inflammatory macrophages.<br>5) Enhance intestinal tight junctions and protect the integrity of the intestinal mucosa.	1) Camptothecin dose-dependently mitigated colitis symptoms, reduced DAI scores and colon tissue pathological damage, and lowered MPO activity and pro-inflammatory mediator levels in DSS-treated mice.<br>2) Camptothecin suppressed the production of multiple pro-inflammatory cytokines and inflammatory-related proteins in LPS-stimulated RAW264.7 macrophages without obvious cytotoxicity within the tested concentration range.	[18]

Table S3. Summary of Saponins anti-CAC by targeting the microbiota-metabolism-immunity.

Chinese medicine	Active compounds	Models	Dose/concentration and route	Pharmacological effects	Mechanisms of action	Main findings	References
Renshen (*Ginseng Radix et Rhizoma*)	Ginsenoside Rg3	SW-480 cells, HT-29 cells, HCT-116 cells, Xenograft tumor model of colon cancer in nude mice	1) In-vivo: ginsenoside Rg3 10, 20, 40 mg/kg, intraperitoneal injection once daily for 3 weeks. 2) In-vitro: ginsenoside Rg3 at 25-200 μM, treated for 48 h.	Anti-tumor/Anti-proliferation effects	1) Down-regulate the mRNA and protein expression levels of C/EBPβ. 2) Inhibit the binding of C/EBPβ and the p65-NFκB subunit in the cell nucleus. 3) Reduce NF-κB-dependent transcriptional activity.	1) Ginsenoside Rg3 suppressed cell proliferation of three human colorectal cancer cell lines in a dose-dependent manner under in-vitro conditions. 2) In the SW480 xenograft model, intraperitoneal Rg3 (10-40 mg/kg/day) significantly suppressed tumor growth in vivo.	[19]
	Ginsenoside C-K	AOM/DSS-induced CAC in Balb/c mice, Validation of the anti-cancer effect of Akkermansia muciniphila on CRC cell lines.	1) In-vivo: ginsenoside compound K (GC-K) was administered via diet feeding at nominal doses of 30 mg/kg and 60 mg/kg; the actual measured dosages were 26.99 mg/kg and 58.65 mg/kg respectively. 2) In-vitro: Akkermansia muciniphila was co-cultured with HCT-116, HT-29 and LOVO CRC cell lines at multiplicity of infection of 100:1 and 1000:1.	Anti-inflammatory, Anti-CRC / Chemopreventive, and Microbiotic Restoration effects.	1) Regulate the balance of intestinal microbial ecology, restore the species richness and evenness of the intestinal microecology, and alleviate the expansion of pathogenic bacteria. 2) Specifically enriched Akkermansia muciniphila. 3) Microbiota-Host Metabolic Interaction. 4) Improve the background of intestinal inflammation and block the cascade progression from chronic colitis to malignant adenoma.	1) High-dose GC-K effectively suppressed tumor growth and relieved intestinal pathological damage in AOM/DSS-treated mice, while low-dose GC-K showed no obvious anti-tumor effect. 2) GC-K partially restored the disturbed gut microbiota structure of model mice, and Akkermansia muciniphila isolated from microbiota could inhibit the proliferation of colorectal cancer cells in vitro.	[20]
	Ginsenoside Rb1	AOM/DSS-induced CAC mice	In-vivo: AOM was injected intraperitoneally at 7.5 mg/kg; 2% DSS drinking water was given in three cycles. Ginsenoside Rb1 was administrated every other day throughout the whole experimental period (18 weeks).	Anti-cancer/chemopreventive, Anti-inflammatory and immunomodulator, and Gut microenvironment restoration effects.	1) Reduce the levels of pro-inflammatory factors such as TNF-α, IL-6, IL-17A, IL-33, IL-1β, and IL-22 that drive "inflammation-to-cancer transformation". 2) Increase the levels of IL-10 to enhance the immune tolerance and anti-inflammatory repair ability of the intestinal mucosa. 3) Reshape the gut microbiota, and through the dual approach of "improving the inflammatory factor microenvironment and regulating the gut microbiota",block the occurrence and development of colorectal cancer.	1) Ginsenoside Rb1 reduced tumor multiplicity and pathological colon injury in AOM/DSS-treated mice, and modulated serum levels of multiple pro-and anti-inflammatory cytokines. 2) Rb1 partly restored the disturbed gut microbiota structure of model mice, and differential gut microbes showed correlation with inflammatory cytokine changes.	[21]

	Ginsenoside derivative AD-1	AOM/DSS-induced CAC mice and CRC xenograft models, in the TAM model, by co-culturing RAW264.7 and CT26 cells.	1) In-vivo: AD-1 was given by oral gavage at 10 mg/kg once daily.<br>2) In-vitro: ginsenoside derivative AD-1 at 2.5, 5, 10 μM for 24 h.	Chemoprevention and Antitumor, Immune microenvironment remodeling, and Anti-chronic colon inflammation effect	1)Promote the phenotypic remodeling and conversion of TAMs from pro-angiogenic, immunosuppressive M2 to tumoricidal M1.<br>2) Inhibit the PI3K/Akt/mTOR signaling network.<br>3) Regulating glycolytic energy metabolism reprogramming.	1) AD-1 alleviated colonic pathological damage and effectively suppressed tumor growth in both AOM/DSS-induced CAC mice and CRC xenograft nude mice.<br>2) AD-1 shifted tumour-associated macrophage phenotypes, increasing M1-like proportion and decreasing M2-like proportion both in-vivo and in-vitro.	[22]
Xiyangshen *Panacis quinquefoli radix*	ginsenosides Rb1	AOM/DSS-induced CAC in A/J mice,	In-vivo: American ginseng (AG) extract was administrated via drinking water at 30 mg/kg/day for consecutive 90 days.	Anti-colitis, Anti-inflammatory, and Anti-proliferation and pro-apoptosis effects.	1)Inhibit the protein levels of a total of 12 pro-inflammatory cytokines in colon tissue, including IL-1α, IL-1β, IL-2, IL-4, IL-6, IL-10, IL-12, IL-17A, IFN-γ, TNF-α, G-CSF, and GM-CSF.<br>2) Inhibition of inflammatory cytokine gene transcription, blocking the continuous activation of inflammatory signals.<br>3) Block cancerous pathways mediated by chronic inflammation.	1) Oral AG extract markedly alleviated DSS-induced colitis, lowered DAI scores and promoted colitis recovery in AOM/DSS-treated mice.<br>2) AG significantly reduced colon tumor number and tumor load in mice, and suppressed elevation of multiple colonic pro-inflammatory cytokines.	[23]
Jiaogulan (Gynostemma pentaphyllum)	triterpenoid saponins	C57BL/6J Apc$^{Min/+}$mice and（WT）C57BL/6J mice.	In-vivo: gynostemma pentaphyllum saponins 500 mg/kg by daily oral gavage for 8 weeks.	Anti-tumor/ Cancer preventive, Prebiotics-like, and Anti-inflammatory effect.	1) Reduced the number of intestinal polyps and the spectrum of sulfate-reducing bacteria.<br>2) Increase the number of beneficial bacteria in the intestine.<br>3) Upregulated E-cadherin, downregulated N-cadherin, and reduced pro-oncogenic β-catenin, p-Src, and p-STAT3.<br>4) Upregulate the anti-inflammatory cytokine IL-4, while downregulating the pro-inflammatory cytokines TNF-β, IL-1β, and IL-18.	1) Preventive gynostemma pentaphyllum saponins treatment significantly reduced the number of intestinal polyps in Apc$^{Min/+}$ mice and reshaped the overall composition of fecal gut microbiota.<br>2) gynostemma pentaphyllum saponins elevated the abundance of beneficial bacteria while suppressing sulfate-reducing bacteria, and improved the intestinal mucosal barrier status of model mice.	[24]
Sanqi *Notoginseng radlx rhizoma*	Panax notoginseng saponins	AOM/DSS-induced CAC mice, HCT-116 cells, HT-29 cells.	1) In-vivo: Panax notoginseng saponins (PNS) via chow supplementation at 30 mg/kg/day and 90 mg/kg/day for the whole experimental period.<br>2) In-vitro: Ginsenoside compound	Anti-inflammatory, Anti-CRC/ Antitumor, and Gut microbiota modulation effects.	1) Reshape the intestinal microecological structure, restore the alpha diversity and richness of the gut microbiota, and enrich Firmicutes, Bacteroidetes, and Verrucomicrobia.<br>2) Upregulate the relative abundance of Akkermansia sp., exerting core anti-inflammatory and anti-cancer regulatory effects, Its main metab	1) Dietary PNS alleviated colitis symptoms, reduced colon tumor load and partially restored the decreased gut microbiota richness and diversity in AOM/DSS-challenged mice.<br>2) PNS increased the relative abundance of	[25]

			K (GC-K) was applied at gradient concentrations to HCT-116 and HT-29 human colorectal cancer cells for 24 h and 48 h.		olic transformation product, ginsenoside compound K (GC-K), can directly inhibit the proliferation of tumor cells such as HT-29, HCT-116.<br>3) Direct anticancer substances produced through gut microbiota-dependent biotransformation.	Akkermansia spp. in model mice, and its gut-microbial metabolite GC-K suppressed the proliferation of HCT-116 and HT-29 cells in vitro.	
		AOM/DSS-induced CAC in C57BL/6 mice, IFN-γ-stimulated Hela cells and CT26 calls	1) In-vivo: Panax notoginseng saponins were given by oral gavage at 75 mg/kg and 150 mg/kg throughout the whole modeling period.<br>2) In-vitro: PNS was applied at gradient concentrations (200-800 μg/mL) to IFN-γ-stimulated Hela cells and CT26-splenocytes co-culture system.	Anti-inflammatory, Chemopreventive/Anti-tumor, and Immune modulation effects.	1) Inhibition of STAT1/IDO1 axis.<br>2) Inhibit Treg cell differentiation and immunosuppression.<br>3) Regulate the tumor immune microenvironment, reduce the accumulation of macrophages ($CD11b^{+}F4/80^{+}$) in colon tissue, decrease the release of the pro-inflammatory factor IL-1β,alleviate chronic inflammation in the colon, and block the transformation from inflammation to tumor.<br>4) The PNS metabolite Rh1 can directly inhibit the enzymatic activity of IDO1, blocking the metabolism of tryptophan to kynurenine.	1)PNS treatment dose-dependently relieved colitis injury and reduced adenoma number, size as well as tumor load in AOM/DSS-challenged mice.<br>2) PNS lowered colonic macrophage infiltration and decreased the proportion of Treg cells in peripheral immune organs and colon tissues of model animals.	[26]
Huangqi *Astragali Radix*	Astragalus saponins	DSS-induced colitis in C57BL/6J mice, HT-29 cells, SW620 cells.	1) In-vivo: AST was administered by oral gavage at 0.1 mg/g body weight for 8 consecutive days.<br>2) In-vitro: AST at gradient concentrations (0.02-0.20 mg/mL) were applied to HT-29 and SW620 human colorectal cancer cells; 50 μg/mL AST was used for follow-up functional cell experiments.	Anti-proliferative, Metabolic regulatory, and Tumor Chemopreventive effect.	1)Suppression of Inflammation-induced Carcinogenesis.<br>2)Inhibition of Aerobic Glycolysis, reduce the activity of key glycolytic enzymes HK2, LDH-A, and Glut-1, decrease glucose uptake and lactate production, downregulate the expression of the oncogene c-Myc, thereby inhibiting the Warburg effect of tumors.<br>3)Induces tumor cell apoptosis, upregulates the expression of pro-apoptotic proteins Bax, Bad, and Bak, and downregulates the expression of anti-apoptotic proteins Bcl-2, pro-caspase3, and PARP.	1) AST suppressed proliferation and triggered apoptosis of HT-29 and SW620 colorectal cancer cells, and lowered cellular glucose uptake and lactate production in vitro.<br>2) AST alleviated weight loss and colon tissue inflammatory injury in DSS-treated mice, and mitigated abnormal aerobic glycolysis changes in colonic tissues.	[27]

	Astragaloside IV	AOM/DSS-induced C57BL/6 mice, LPS-stimulated rat small intestinal epithelial cells (IEC-6 cells).	1) In-vivo: astragaloside-IV was orally gavaged at 20, 40, 80 mg/kg/d throughout the experiment; GW9662 (1 mg/kg/d) was given by intraperitoneal injection for pathway-blocking validation.<br>2) In-vitro: rat intestinal epithelial IEC-6 cells were stimulated by LPS, then treated with astragaloside-IV at 5, 20, 50 μM.	Anti-tumor, Anti-inflammatory, Antioxidant, and DNA Protection effects.	1) Activation of PPARγ Signaling Axis, Promote the expression of Nrf2 and HO-1, restore the levels of SOD, CAT, and GSH, inhibit the expression of iNOS and reduce NO production in the intestine and IEC-6 cells, thereby reducing DNA damage caused by intestinal inflammation and thus inhibiting the occurrence of colon tumors.<br>2) Inhibit colon shortening, prevent intestinal mucosal damage, reduce the number of tumors and Ki67 protein expression.<br>3) The novelty lies in targeting PPARγ to inhibit DNA damage, in order to prevent the occurrence of CAC.	1) Astragaloside-IV dose-dependently mitigated colonic pathological injury and reduced tumor number in AOM/DSS-treated mice, and improved peripheral blood and immune-organ abnormalities.<br>2) Astragaloside-IV alleviated inflammatory-related oxidative stress and DNA damage both in CAC mouse colon tissue and LPS-challenged IEC-6 intestinal epithelial cells.	[28]
Jiegeng *Platycodonis radix*	Platycodin D	DSS-induced ulcerative colitis in C57BL/6 mice, THP-1 cell, PMA-induced differentiation and LPS+ATP inflammation induction, PLD gradient concentration treatment.	1) In-vivo: PLD was orally gavaged at 7.5 mg/kg once daily for 7 days; AAV9 virus was injected via a tail vein to achieve ZNF70 gene knockdown.<br>2) In-vitro: PLD at 1, 3, 10 μM, followed by LPS plus ATP stimulation.	Anti-inflammatory, Mucosal protection and repair, and Systemic safety effects.	1) By downregulating the expression of the transcription factor ZNF70, targeting the regulation of ZNF70.<br>2) Reduce the protein expression of NLRP3, ASC, and cleaved caspase-1, and inhibit the conversion of Pro-IL-1β to active IL-1β.<br>3) Block STAT3/Src signaling.<br>4) Through the synergistic effects of multiple targets and multiple pathways, break the vicious cycle of the intestinal inflammatory microenvironment.	1) PLD alleviated DSS-triggered colitis symptoms in mice, improved colon pathological injury and reduced the secretion of serum pro-inflammatory factor IL-1β.<br>2) PLD suppressed inflammatory mediator release in LPS-ATP-stimulated THP-1 macrophages, and displayed good biosafety without obvious organ toxicity in vivo.	[29]
Baitouweng *Pulsatillae radix*	Anemoside B4	AOM/DSS-induced CAC mice.	In-vivo: anemoside B4 was administered by intraperitoneal injection at 20 mg/kg once daily throughout the modeling period.	Chemopreventive and Anti-tumor, Anti-inflammatory, and Mucosal protection effects.	1) Inhibition of the Wnt/β-catenin signaling pathway, Reduce the occurrence and progression of colon inflammation-related cancers.<br>2) Reduce the mRNA and protein expression levels of Wnt6.<br>3) Block core transcription co-activators and inhibit the expression of β-catenin.<br>4) Inhibit the transcription of downstream target genes and proteins, thereby suppressing the uncontrolled proliferation of cancer cells and the process of inflammation-to-cancer transformation.	1) Anemoside B4 reduced colon tumor burden and down-regulated the elevated colonic pro-inflammatory cytokine levels in CAC model animals.<br>2) Anemoside B4 alleviated clinical symptoms and colon pathological injury in AOM/DSS-treated CAC mice.	[30]

		AOM/DSS-induced CAC in C57BL/6 mice.	In-vivo: anemoside B4 was intraperitoneally injected daily at 10, 20, 40 mg/kg throughout the modeling period.	Anti-tumor, Mucosal protection, and Metabolic regulation effects.	1)Downregulate the expression of the key transcription factor SREBP-1 and its downstream rate-limiting enzymes FASN, ACC α, and SCD-1, thereby inhibiting de novo fatty acid synthesis.<br>2) Downregulation of the key transcription factor PPARα and its regulated CPT-1 and ACOX expression inhibits fatty acid β-oxidation.<br>3) Downregulate the expression of UCP-2, involved in regulating lipid metabolism balance and oxidative stress response in mitochondria.<br>4) Blocking the supply of raw materials for biofilm construction.	1) Anemoside B4 alleviated colonic inflammatory injury, improved colon shortening and reduced colon tumour burden in AOM/DSS-challenged CAC mice in a dose-related manner.<br>2) anemoside B4 decreased the abundance of fatty acids, phospholipids and carnitine metabolites in colon tumour tissues of model animals.	[31]
Chonglou *Paridid rhizoma*	Paris saponin VII	HT-29 cells, SW-620 cells, Murine model of xenograft tumor, DMH/DSS-induced CAC in ICR mice.	1) In-vivo: PSVII was given by intraperitoneal injection at 2.5, 5, 10 mg/kg every three days.<br>2) In-vitro: PSVII was applied to HT-29 and SW-620 human colorectal cancer cells at gradient concentrations; IC_{50} values were 1.02 μM for HT-29 and 4.90 μM for SW-620	Anti-tumor proliferation, Chemoprevention of cancer, and Intestinal protection effects.	1) Downregulate Ras activity, inhibit the PI3K/AKT/GSK-3βpathway and reduce the phosphorylation levels of AKT and GSK-3β, and inhibit the MAPK pathway, reduce the phosphorylation levels of MEK1/2 and ERK1/2, and block pathway activation.<br>2) Activate the endogenous apoptosis pathway, upregulate the expression of cleaved-caspase-3, cleaved-caspase-9, Bax, and PARP cleavage products, and downregulate the anti-apoptotic protein Bcl-2.<br>3) Inducing G1 phase arrest, Prevent its DNA replication and division.	1) PSVII concentration-dependently suppressed the viability of HT-29 and SW-620 colorectal cancer cells, and triggered cell apoptosis together with G_1-phase cell-cycle arrest in vitro.<br>2) PSVII alleviated intestinal pathological injury and reduced tumour incidence in DMH/DSS-treated CAC mice, and markedly shrank tumour volume in SW-620 xenograft nude mice.	[32]

Table S4. Summary of Polysaccharides anti-CAC by targeting the microbiota-metabolism-immunity.

Chinese medicine	Active compounds	Models	Dose/concentration and route	Pharmacological effects	Mechanisms of action	Main findings	References
Lingzhi *Ganoderma*	GLP	AOM/DSS-induced CAC in C57BL/6 mice, RAW264.7, HT-29 and NCM460 cells.	1) In-vivo: GLP was administrated by intragastric gavage at 200 mg/kg and 300 mg/kg once per day. 2) In-vitro: GLP was applied to RAW264.7, HT-29 and NCM460 cells at gradient concentrations ranging from 0.2-3.2 mg/mL with LPS stimulation	Anti-tumor, Anti-inflammatory, Mucosal barrier protection, and Microbiota modulation effects.	1)Inhibiting the TLR4/MyD88/NF-κB signaling pathway alleviated microbiota dysbiosis induced by AOM/DSS and increased SCFA production. 2) Reshape the intestinal microbiota structure, reduce the abundance of pathogenic bacteria, and increase the proportion of beneficial bacteria such as SCFA producing probiotics. 3) Inhibit macrophage infiltration and downregulate the expression of IL-1β, iNOS, and COX-2. 4) Regulates the body's immune and metabolic homeostasis, modulates innate immune responses through the TLR4 pathway, and inhibits tumor metabolism to slow abnormal cancer cell proliferation.	1) GLP alleviated colitis-related clinical manifestations and colon pathological injury, and dose-dependently reduced tumor number and size in AOM/DSS-treated CAC mice. 2) GLP reshaped the disturbed gut microbiota composition, elevated fecal short-chain fatty acid levels and improved intestinal barrier function in model animals.	[33]
Baizhu *Atractylodis macrocephalae rhizoma*	PAMK	DSS-induced UC in C57BL/6 mice.	In-vivo: PAMK was administrated by intragastric gavage at 100, 200, 400 mg/kg once daily.	Anti-colitis, Immunomodulatory, and Mucosal protective effect.	1) Regulate the abnormal Th17/Treg cell imbalance in UC, inhibit excessive adaptive immune responses, and alleviate colonic immune inflammation. 2) Downregulate the mRNA expression of IL-6- in colon tissue, inhibit the phosphorylation activation of STAT3 protein, reduce the p-STAT3/STAT3 ratio, and block IL-6-mediated STAT3 signaling. 3) Downregulate the expression of TNF-α, IL-1β, IL-18, and IL-23, and reduce MPO activity in colon tissue. 4) Upregulate the expression of intestinal tight junction proteins ZO-1 and Occludin, repair DSS-induced intestinal barrier structural damage, and reduce intestinal mucosal permeability.	1) PAMK reduced weight loss, DAI score and colon pathological damage in DSS-exposed mice, and exerted the most obvious protective effect at 200 mg/kg among tested doses. 2) PAMK lowered colonic neutrophil infiltration and pro-inflammatory cytokine levels, and elevated the expression of intestinal tight-junction proteins to maintain epithelial barrier integrity.	[34]

Fuling<br>*Poria*	Poria cocos polysaccharides	AOM/DSS-induced CAC in C57BL/6 mice.	In-vivo: PCP were given by oral gavage at 200 mg/kg per day throughout the experiment.	Chemical prevention, Anti-inflammatory, and Microbiota-remodeling effects.	1) Remodeling intestinal microbiota, Increase the relative abundance of beneficial bacteria and reduce the proportion of pathogens associated with inflammation and tumors.<br>2) Restoring intestinal barrier, promote mucosal Iayer repair and the expression of tight junction proteins, and reduce intestinal permeability.<br>3) Modulates host immunity and metabolism, downregulates pro-inflammatory cytokines TNF-α, IL-6, IL-1β, and upregulates anti-inflammatory cytokine IL-10.	1) PCP reduced tumour incidence, tumour number and maximum tumour size in AOM/DSS-treated mice, and alleviated colonic pathological injury without obvious toxicity to major organs.<br>2) PCP reshaped the structure of intestinal microbiota, improved intestinal barrier status and lowered serum pro-inflammatory cytokine levels in model mice, which was further supported by fecal microbiota transplantation results.	[35]
Huangqi<br>*Astragali Radix*	APS	AOM/DSS-induced CAC in C57BL/6 mice, $CD8^+$ T cells, MC38 calls.	1) In-vivo: model and APS-treated groups received 12.5 mg/kg AOM via intra-peritoneal injection, APS was orally gavaged at 100 mg/kg, 200 mg/kg and 400 mg/kg every two days throughout the experiment.<br>2) In-vitro: APS at gradient concentrations (100, 250, 500, 750 μg/mL) was applied to MC38 colon cancer cells co-cultured with isolated mouse $CD8^+$ T cells.	Immune regulation, Anti-tumor, and, Anti-inflammatory effects.	1)Targeted regulation of the STAT3/Gal-3/LAG3 signaling pathway<br>2) Block immune checkpoints and reverse T cell exhaustion, relieve the suppression of effector T cells by the tumor microenvironment, and restore the cytotoxic killing function of CD8 T cells.<br>3) Multi-target network synergistic regulation, showing the synergistic therapeutic characteristics of traditional Chinese medicine multiple components, multiple targets, multiple pathways	1) APS restrained colorectal cancer progression in AOM/DSS-treated mice and improved the effector function of tumor-infiltrating $CD8^+$ T cells in vivo.<br>2) In the MC38-$CD8^+$ T co-culture system, high-concentration APS suppressed MC38 cell viability and relieved the functional exhaustion status of co-cultured $CD8^+$ T cells.	[36]
		DSS (molecular weight 36-50 kDa) induced UC in BALB/c mice.	In-vivo: APS were administrated by oral gavage at 200 and 400 mg/kg.	Anti-inflammatory, Antioxidant, Intestinal barrier protection, and Prebiotic-like effect.	1) Regulate the microbiota-metabolism axis, restoring the synthesis of SCFAs such as acetate, propionate, and butyrate in the intestine.<br>2) Correct Th17/Treg immune imbalance through SCFA-mediated signal transduction.<br>3) Inhibit the JAK/STAT signaling pathway, thereby inhibiting the expression of key transcription factors mediating Th17 differentiation.<br>4) Block the NF-κB inflammatory pathway and reduce the transcription and release of inflammatory factors.<br>5) Regulate FFARs/GPRs receptors and participate in epigenetic regulation of immune respon	1) APS alleviated colitis-related clinical manifestations and colon histopathological injury, improved intestinal mucosal barrier function in DSS-challenged mice.<br>2) APS reshaped gut microbiota composition and elevated short-chain fatty-acid levels; the protective effects of APS were largely abolished after gut microbiota depletion, which was further supported by fecal microbiota transplantation results.	[37]

					ses.		
Renshen Ginseng Radix et Rhizoma	GPS	AOM/DSS-induced CAC in C57BL/6J mice, MC38-induced CRC in C57BL/6J mice.	In-vivo: GPS were orally administered by gavage at 100, 200, 400 mg/kg every three days.	Anti-tumor, Immune regulation, Metabolic regulation, and Prebiotic effect.	1) Targetedly regulate the gut microecology, promote the proliferation of Lachnospiraceae bacterium, and correct the dysbiotic state of the gut microbiota in model mice.<br>2) Drive SCFAs metabolism, increase the level of SCFAs in the gut by enriching acid-producing bacteria.<br>3) Inhibition of MDSCs, reduce the levels of PMN-MDSCs and M-MDSCs in tumor tissues.<br>4) Enhance CD8 T cell immune effector function.	1) GPS oral treatment significantly alleviated colorectal tumorigenesis in two mouse models, and its anti-tumour effect reached saturation at 200 mg/kg without further enhancement at higher dosage.<br>2) Fecal microbiota transplantation or direct *Lachnospiraceae* bacterium intervention reproduced part of the anti-CRC effect of GPS, and combined GPS-*Lachnospiraceae* bacterium treatment achieved the strongest tumour-suppressing outcome.	[38]
Gancao *Glycyrrhizae radix et rhizoma*	GPP	DSS-induced UC in BALB/c mice	In-vivo: GPP was orally administrated by gavage at doses of 100, 200, 300 mg/kg.	Anti-inflammatory, Barrier protection, Microbiota modulation, and Immunomodulatory effects.	1) Reduce colon damage, strengthen the physical barrier of the intestinal epithelium, and protect the integrity of the intestinal barrier.<br>2) Regulate the abundance of specific gut microbiota to reverse inflammation induced microecological imbalance.<br>3) Upregulate the expression of the anti-inflammatory factor IL-10, downregulate the secretion of the pro-inflammatory factors TNF-α and IL-6, and correct the imbalance of intestinal inflammatory factors.<br>4) Regulate the Th17/Treg cell balance, maintain local immune tolerance, and restore intestinal immune homeostasis.	1) GPP intervention relieved clinical and pathological manifestations of DSS-triggered colitis in mice, and the 200 mg/kg medium-dose group achieved the optimal therapeutic effect among three tested dosages.<br>2) GPP partially restored gut microbiota disturbance in colitis mice, with observable improvements on multiple bacterial taxa at phylum and genus levels.	[39]

Xianggu *Lentinula E dodes*	lentinan	DSS-induce d colitis in C57BL/6 mice, TNB S-induced colitis in B ALB/c mic e, AOM/D SS-induced CAC in B ALB/c mic e, RAW26 4.7 cells, Caco-2cells.	1) In-vivo: Lenti nan was administ rated to mice at 5, 10, 20 mg/kg. 2) In-vitro: treate d with lentinan a t 0.5-2 mg/ML.	Anti-inflammat ory, Signal pat hway regulatio n, Anti-colorect al cancer, and Mucosal protec tion effects.	1) Inhibit the TLR4/NF-κB in flammatory signaling pathway, regulate the expression of dow nstream IL-13, CD30L, and bl ock the recruitment of neutrop hils and inflammatory cells. 2) Regulate the tumor necrosis factor receptor pathway and in hibit the malignant transformati on of colonic epithelial cells. 3) Maintain intestinal immune balance, regulate the polarizatio n balance of Th1/Th2 cytokine s, and alleviate T cell-mediated immunopathological damage. 4) Control chronic inflammator y states and reduce gene mutat ions and abnormal cell prolifer ation caused by inflammatory s timuli.	1) Lentinan alleviated pathological injury in multiple colitis mouse models in a dose-de pendent manner, and high-dose lentinan sho wed better efficacy th an positive-control SA SP. 2) Lentinan reduced t umour burden in AO M/DSS-induced CAC mice and partially res tored the disturbed gu t microbiota compositi on of colitis animals.	[40]
Gouqizi *Lycii fructus*	LBP	DSS-induce d colitis in C57BL/6 mice,.	In-vivo: LBP wa s intragastrically administered at 1 50 mg/kg and 3 00 mg/kg.	Anti-inflammat ory, barrier pro tection, microe cological regula tion, and anti-t umor effects.	1) Reshape the gut microbiota structure. 2) Promote the gut microbiota to produce higher levels of SC FAs, activating epithelial GPR4 3/GPR109a receptors. 3) Upregulate the transcription and expression of ZO-1, Occlu din, and Claudin-1 through the TGR5 pathway. 4) Downregulate the IL-6/STA T3, TLR4/NF-κB, and NLRP3 inflammasome signaling pathw ays, and improve Th17/Treg i mmune imbalance.	1) LBP alleviated DS S-triggered colitis-relat ed clinical and histolo gical manifestations in mice, and high-dose LBP exhibited superio r protective effects co mpared with the low-dose group. 2) LBP reshaped the disturbed gut microbi ota composition of co litis mice and exerted comparable therapeut ic performance to me salazine in many obse rvation indicators.	[41]
Shengjiang *Zingiberis r hizome rece ns*	ZOP-1	DSS-induce d UC in C 57BL/6 mi ce.	In-vivo: ZOP-1 was orally admin istered by gavage at 500 mg/kg an d 1000 mg/kg.	Anti-inflammat ory, antioxidan t, barrier prote ction, and imm une regulation effects.	1) Repair intestinal barrier inte grity. 2) Correct dysbiosis caused by UC and restore the balance bet ween beneficial and pathogenic bacteria 3) Inhibit the TLR4/NF-κB/M APK signaling pathway, downr egulate the expression of TLR4 and MyD88, reduce the phosp horylation activation of NF-κ B and P38, and block the dow nstream inflammatory cascade. 4) Reshape the microbiota, reg ulate microbiota-driven SCFAs production, and maintain the h omeostasis of the intestinal mic roenvironment.	1) ZOP-1 alleviated macroscopic and histo pathological colon inj uries in colitis mice i n a dose-dependent m anner, and high-dose ZOP-1 achieved thera peutic effects compara ble to SASP. 2) ZOP-1 improved multiple disease-relate d observation indicato rs of colitis mice, an d restored the overall disturbed structure of the intestinal microbio ta.	[42]

Yuxingcao<br>*Houttuyniae herba*	HCPs	DSS-induced UC in C57BL/6 mice, IEC-6 cells, Ana-1 cells.	1)In-vivo: HCPs were intragastrically administered at 40 mg/kg and 80 mg/kg.<br>2) In-vitro: treated with HCPs at concentrations ranging from 1-100 μg/mL.	Relieve clinical symptoms, intestinal barrier protection, anti-inflammatory and immune regulation effects.	1) Upregulate the expression of tight junction protein ZO-1, promote goblet cells to secrete mucin MUC2, and inhibit apoptosis of intestinal epithelial cells.<br>2) Inhibit pro-inflammatory M1 macrophage polarization and induce anti-inflammatory M2 macrophage expansion.<br>3) Reshape Th17/Treg balance, correct immune imbalance.<br>4) Inhibit the mRNA expression of TNF-α, IL-6, and IL-1β, and increase the level of the anti-inflammatory factor IL-10.<br>5) Reverse DSS-induced microecological imbalance and reconstruct the structure of symbiotic bacteria to help maintain homeostasis.	1) HCPs relieved colitis-related clinical manifestations and colon pathological damage in DSS-challenged mice, and the 80 mg/kg high-dose group exhibited more obvious protective effects.<br>2) HCPs improved survival status of oxidative-damaged intestinal epithelial cells and reversed the gut microbiota compositional disturbance caused by DSS induction.	[43]
Wuweizi<br>*Schisandrae chinensis fructus*	SCP	DSS-induced UC in C57BL/6 mice.	In-vivo: SCP was orally given by gavage at 8 g/kg daily.	Anti-inflammatory and immune regulation, antioxidative stress, regulation of intestinal microecology, and microbial metabolism promotes growth effects.	1) Regulate the network of inflammatory cytokines<br>2) Reduce the levels of MPO, NO, ROS, and MDA in local colonic lesions, and increase the levels of GSH and SOD.<br>3) Reshape the composition and diversity of the gut microbiota, and restore the balance of the gut microecology.<br>4) Drive SCFAs metabolic cascade, regulate host immune response, inhibit the release of inflammatory factors, and maintain the function of the intestinal mucosal barrier.	1) SCP alleviated DSS-triggered colitis-related weight loss and colon histopathological injury in mice, and exhibited better therapeutic performance than SASP in multiple detected indicators.<br>2) SCP improved the disturbed gut microbial community structure of colitis mice and elevated the intestinal short-chain fatty acid contents in model animals.	[44]
Tiepishihu<br>*Dendrobii officinalis caulis*	DOP	DSS-induced UC in C57BL/6 mice.	In-vivo: Dendrobium officinale leaf polysaccharide (50, 75, 100 mg/kg) and stem polysaccharide (150, 200, 250 mg/kg) were intragastrically administrated daily for 14 days.	Anti-inflammatory, barrier repair, biofilm and microbial regulation, and metabolic homeostasis regulation effects.	1) Repair the intestinal physical and physiological barriers, and inhibit subsequent inflammatory cascade reactions.<br>2) Reshape the intestinal microbiota structure, promote the suppression of pathogenic bacteria, and enrich probiotics.<br>3) Correct purine metabolism disorders, maintain intestinal mucosal immune homeostasis, and block excessive production of inflammatory mediators.<br>4) Multi-site synergistic regulation, combined with gut microbiota remodeling and intestinal barrier repair, alleviates DSS-induced UC from multiple dimensions.	1) Both DOPY and DOPY-J relieved colitis-related clinical manifestations and colon pathological injuries in mice, with DOPY exerting good effects at low dose while DOPJ showing better performance at high dose.<br>2) DOP intervention reshaped the altered gut microbiota profile of colitis mice and produced obvious regulatory effects on cecal metabolic profiles including purine-related metabolites.	[45]

References

1. Li, Z.; Lin, M.; Li, Y.; Shao, J.; Huang, R.; Qiu, Y.; Liu, Y.; Chen, L. Total flavonoids of Sophora flavescens and kurarinone ameliorated ulcerative colitis by regulating Th17/Treg cell homeostasis. *J Ethnopharmacol* **2022**, *297*, 115500, doi:10.1016/j.jep.2022.115500.

2. Li, C.; Wang, L.; Zhao, J.; Wei, Y.; Zhai, S.; Tan, M.; Guan, K.; Huang, Z.; Chen, C. Lonicera rupicola Hook.f.et Thoms flavonoids ameliorated dysregulated inflammatory responses, intestinal barrier, and gut microbiome in ulcerative colitis via PI3K/AKT pathway. Phytomedicine 2022, 104, 154284, doi:10.1016/j.phymed.2022.154284.
3. Wu, Z.; Liu, W.; Wang, M.; Gebre, B.A.; Mekonnen, S.A.; Huang, W.; Sui, Z.; Corke, H. Phenolic Extracts of Hulless Barley Grass Alleviate Progression of Colitis-Related Colorectal Cancer by Inhibiting the Overexpression of Wnt/beta-Catenin Signaling Pathway. J Food Sci 2025, 90, e70681, doi:10.1111/1750-3841.70681.
4. Yue, S.; Qin, Y.; Kang, A.; Tao, H.; Zhou, G. Total Flavonoids of Glycyrrhiza uralensis Alleviates Irinotecan-Induced Colitis via Modification of Gut Microbiota and Fecal Metabolism; frontiersin.org: 2021.
5. Wu, M.; Wu, Y.; Deng, B.; Li, J.; Cao, H.; Qu, Y.; Qian, X.; Zhong, G. Isoliquiritigenin decreases the incidence of colitis-associated colorectal cancer by modulating the intestinal microbiota. Oncotarget 2016, 7, 85318-85331, doi:10.18632/oncotarget.13347.
6. Liu, M.; Yang, C.; Peng, X.; Zheng, S.; He, H.; Wang, W.; Li, Y. Formononetin suppresses colitis-associated colon cancer by targeting lipid synthesis and mTORC2/Akt signaling. Phytomedicine 2025, 142, 156665, doi:10.1016/j.phymed.2025.156665.
7. Chen, Y.; Qi, Y.; Jiang, Y.; Li, Y.; Yang, S.; Wang, L.; Li, M.; Chai, K.; Wang, Y. Icariin modulates the tumor microenvironment in colorectal cancer by targeting M2 macrophage polarization via PI3K/AKT pathway. Bioorg Med Chem 2025, 129, 118317, doi:10.1016/j.bmc.2025.118317.
8. Cui, L.; Feng, L.; Zhang, Z.H.; Jia, X.B. The anti-inflammation effect of baicalin on experimental colitis through inhibiting TLR4/NF-kappaB pathway activation. Int Immunopharmacol 2014, 23, 294-303, doi:10.1016/j.intimp.2014.09.005.
9. Zhu, L.; Xu, L.; Zhao, S.; Shen, Z.; Shen, H. Protective effect of baicalin on the regulation of Treg/Th17 balance, gut microbiota and short-chain fatty acids in rats with ulcerative colitis. Applied Microbiology and … 2020, doi:10.1007/s00253-020-10527-w.
10. Yan, S.; Chang, J.; Hao, X.; Liu, J.; Tan, X.; Geng, Z.; Wang, Z. Berberine regulates short-chain fatty acid metabolism and alleviates the colitis-associated colorectal tumorigenesis through remodeling intestinal flora. Phytomedicine 2022, 102, 154217, doi:10.1016/j.phymed.2022.154217.
11. Wang, M.; Ma, Y.; Yu, G.; Zeng, B.; Yang, W.; Huang, C.; Dong, Y.; Tang, B.; Wu, Z. Integration of microbiome, metabolomics and transcriptome for in-depth understanding of berberine attenuates AOM/DSS-induced colitis-associated colorectal cancer. Biomedicine & Pharmacotherapy 2024, 179, doi:10.1016/j.biopha.2024.117292.
12. Deng, J.; Zhao, L.; Yuan, X.; Li, Y.; Shi, J.; Zhang, H.; Zhao, Y.; Han, L.; Wang, H.; Yan, Y.; et al. Pre-Administration of Berberine Exerts Chemopreventive Effects in AOM/DSS-Induced Colitis-Associated Carcinogenesis Mice via Modulating Inflammation and Intestinal Microbiota. Nutrients 2022, 14, doi:10.3390/nu14040726.
13. Li, C.; Dong, N.; Wu, B.; Mo, Z.; Xie, J.; Lu, Q. Dihydroberberine, an isoquinoline alkaloid, exhibits protective effect against dextran sulfate sodium-induced ulcerative colitis in mice. Phytomedicine 2021, 90, 153631, doi:10.1016/j.phymed.2021.153631.
14. Zhang, Y.; Zhang, Y.; Zhao, Y.; Wu, W.; ... Protection against ulcerative colitis and colorectal cancer by evodiamine via anti-inflammatory effects; spandidos-publications.com: 2022.
15. Wang, M.; Zhou, B.; Cong, W.; Zhang, M.; Li, Z.; Li, Y. Amelioration of AOM/DSS-Induced Murine Colitis-Associated Cancer by Evodiamine Intervention is Primarily Associated with Gut Microbiota-Metabolism-Inflammatory Signaling Axis; frontiersin.org: 2021.
16. Yao, H.; Shi, Y.; Yuan, J.; Sa, R.; Chen, W.; Wan, X. Matrine protects against DSS-induced murine colitis by improving gut barrier integrity, inhibiting the PPAR-α signaling pathway, and modulating gut microbiota. Int Immunopharmacol 2021, 100, 108091, doi:10.1016/j.intimp.2021.108091.
17. Liu, M.; Liu, F.; Pan, Y.; Xiong, Y.; Zeng, X.; Zheng, L. Oxymatrine ameliorated experimental colitis via mechanisms involving inflammatory DCs, gut microbiota and TLR/NF-κB pathway. International … 2023.
18. Wang, Y.; Liu, K.; Qi, Z.; Chen, T.; Yu, W.; Jiang, Y.; Li, G.; Xiao, H. Therapeutic Mechanism and Effect of Camptothecin on Dextran Sodium Sulfate-Induced Ulcerative Colitis in Mice. J Immunol Res 2021, 2021, 5556659, doi:10.1155/2021/5556659.
19. Yang, X.; Zou, J.; Cai, H.; Huang, X.; Yang, X.; Guo, D.; Cao, Y. Ginsenoside Rg3 inhibits colorectal tumor growth via down-regulation of C/EBPβ/NF-κB signaling. Biomedicine & Pharmacotherapy 2017, 96, 1240-1245, doi:10.1016/j.biopha.2017.11.092.
20. Shao, L.; Guo, Y.; Wang, L.; Chen, M.; ... Effects of ginsenoside compound K on colitis-associated colorectal cancer and gut microbiota

profiles in mice; pmc.ncbi.nlm.nih.gov: 2022.

21. Wang, L.; Zhang, Q.Q.; Xu, Y.Y.; Zhang, R.; Zhao, Q.; Zhang, Y.Q.; Huang, X.H.; Jiang, B.; Ni, M. Ginsenoside Rb1 Suppresses AOM/DSS-induced Colon Carcinogenesis. Anticancer Agents Med Chem 2023, 23, 1067-1073, doi:10.2174/1871520623666230119092735.

22. Fu, Y.; Zhang, J.; Fang, X.; An, Y.; Huang, S.; Feng, X.; Jiang, X.; Chen, Z.; Quan, X.; Jin, D.; et al. Ginsenoside derivative AD-1 suppresses colitis-associated colorectal cancer progression by reprogramming tumor-associated macrophage polarization via the AMPK/mTOR-glycolysis pathway. Bioorg Chem 2026, 169, 109458, doi:10.1016/j.bioorg.2025.109458.

23. Yu, C.; Wen, X.D.; Zhang, Z.; Zhang, C.F.; Wu, X.H.; Martin, A.; Du, W.; He, T.C.; Wang, C.Z.; Yuan, C.S. American ginseng attenuates azoxymethane/dextran sodium sulfate-induced colon carcinogenesis in mice. J Ginseng Res 2015, 39, 14-21, doi:10.1016/j.jgr.2014.07.001.

24. Chen, L.; Brar, M.S.; Leung, F.C.; Hsiao, W.L. Triterpenoid herbal saponins enhance beneficial bacteria, decrease sulfate-reducing bacteria, modulate inflammatory intestinal microenvironment and exert cancer preventive effects in ApcMin/+ mice. Oncotarget 2016, 7, 31226-31242, doi:10.18632/oncotarget.8886.

25. Chen, L.; Chen, M.Y.; Shao, L.; Zhang, W.; Rao, T.; Zhou, H.H.; Huang, W.H. Panax notoginseng saponins prevent colitis-associated colorectal cancer development: the role of gut microbiota. Chin J Nat Med 2020, 18, 500-507, doi:10.1016/S1875-5364(20)30060-1.

26. Li, X.M.; Yuan, D.Y.; Liu, Y.H.; Zhu, L.; Qin, H.K.; Yang, Y.B.; Li, Y.; Yan, F.; Wang, Y.J. Panax notoginseng saponins prevent colitis-associated colorectal cancer via inhibition IDO1 mediated immune regulation. Chin J Nat Med 2022, 20, 258-269, doi:10.1016/S1875-5364(22)60179-1.

27. Guo, H.; Wan, B.; Wang, J.; Zhang, J.; Yao, W.; Shen, Z. Astragalus saponins inhibit cell growth, aerobic glycolysis and attenuate the inflammatory response in a DSS-induced colitis model. Int J Mol Med 2019, 43, 1041-1048, doi:10.3892/ijmm.2018.4036.

28. Liang, J.; Yang, C.; Li, P.; Zhang, M.; Xie, X.; Xie, X.; Chen, Y.; Wang, Q.; Zhou, L.; Luo, X. Astragaloside IV inhibits AOM/DSS-induced colitis-associated tumorigenesis via activation of PPARgamma signaling in mice. Phytomedicine 2023, 121, 155116, doi:10.1016/j.phymed.2023.155116.

29. Han, J.; Tai, Y.; Cao, S.; Xu, R.; Liu, X.; Cao, S.; Xing, Y.; Li, M.; Zuo, H.; Ma, J.; Jin, X. Platycodin D targets ZNF70 to inhibit NLRP3 inflammasome and STAT3 signaling pathway to ameliorate colitis. Int Immunopharmacol 2025, 165, 115422, doi:10.1016/j.intimp.2025.115422.

30. Niu, X.; Hou, S.; Yu, Y.; Liu, C.; Yang, Y.; Luo, Y.; Yan, X. Anemoside B4 alleviates colitis-associated colon cancer progression via Wnt/beta-catenin signaling pathway. Eur J Pharmacol 2026, 1011, 178428, doi:10.1016/j.ejphar.2025.178428.

31. Yang, X.; Jia, J.; Xie, X.X.; Wan, M.Q.; Feng, Y.L.; Luo, Y.Y.; Ouyang, H.; Yu, J. [Anemoside B4 regulates fatty acid metabolism reprogramming in mice with colitis-associated cancer]. Zhongguo Zhong Yao Za Zhi 2023, 48, 2325-2333, doi:10.19540/j.cnki.cjcmm.20221122.402.

32. Li, Y.; Sun, Y.; Fan, L.; Zhang, F.; Meng, J.; Han, J.; Guo, X.; Zhang, D.; Zhang, R.; Yue, Z.; Mei, Q. Paris saponin VII inhibits growth of colorectal cancer cells through Ras signaling pathway. Biochem Pharmacol 2014, 88, 150-157, doi:10.1016/j.bcp.2014.01.018.

33. Guo, C.; Guo, D.; Fang, L.; Sang, T.; Wu, J.; Guo, C.; Wang, Y.; Wang, Y.; Chen, C.; Chen, J.; et al. Ganoderma lucidum polysaccharide modulates gut microbiota and immune cell function to inhibit inflammation and tumorigenesis in colon. Carbohydr Polym 2021, 267, 118231, doi:10.1016/j.carbpol.2021.118231.

34. Yang, M.; Zhang, Q.; Taha, R.; Abdelmotalab, M. Polysaccharide from Atractylodes macrocephala Koidz. ameliorates DSS-induced colitis in mice by regulating the Th17/Treg cell balance; frontiersin.org: 2022.

35. Chen, L.; Zhao, S.; Chen, Q.; Luo, P.; Li, X.; Song, Y.; Pan, S.; Wu, Q.; Zhang, Y.; Shen, X.; Chen, Y. Poria cocos polysaccharides ameliorate AOM/DSS-induced colorectal cancer in mice by remodeling intestinal microbiota composition and enhancing intestinal barrier function. Int J Biol Macromol 2025, 315, 144477, doi:10.1016/j.ijbiomac.2025.144477.

36. Li, Q.; Zhang, C.; Xu, G.; Shang, X.; Nan, X.; Li, Y.; Liu, J.; Hong, Y.; Wang, Q.; Peng, G. Astragalus polysaccharide ameliorates CD8(+) T cell dysfunction through STAT3/Gal-3/LAG3 pathway in inflammation-induced colorectal cancer. Biomed Pharmacother 2024, 171, 116172, doi:10.1016/j.biopha.2024.116172.

37. Zhang, Y.; Ji, W.; Qin, H.; Chen, Z.; Zhou, Y.; Zhou, Z.; Wang, J.; Wang, K. Astragalus polysaccharides alleviate DSS-induced ulcerative colitis in mice by restoring SCFA production and regulating Th17/Treg cell homeostasis in a microbiota-dependent manner. Carbohydr Polym 2025, 349, 122829, doi:10.1016/j.carbpol.2024.122829.
38. Zong, G.; Deng, R.; Pan, Y.; Liu, M.; Zhu, H.; Tao, R.; Shan, Y.; Wei, Z.; Lu, Y. Ginseng polysaccharides ameliorate colorectal tumorigenesis through Lachnospiraceae-mediated immune modulation. Int J Biol Macromol 2025, 307, 142015, doi:10.1016/j.ijbiomac.2025.142015.
39. Zeng, D. Glycyrrhiza pallidiflora Polysaccharide Ameliorates DSS-Induced Colitis by Protecting Intestinal Barrier Integrity; 2025; Volume 83, pp. 3919-3932.
40. Liu, Y.; Zhao, J.; Zhao, Y.; Zong, S.; Tian, Y.; Chen, S.; Li, M.; Liu, H.; Zhang, Q.; Jing, X.; et al. Therapeutic effects of lentinan on inflammatory bowel disease and colitis-associated cancer. J Cell Mol Med 2019, 23, 750-760, doi:10.1111/jcmm.13897.
41. Li, R.; Yang, P.; Liu, B.; Ye, Z.; Zhang, P.; Li, M.; Gong, Y.; Huang, Y.; Yang, L.; Li, M. Lycium barbarum polysaccharide remodels colon inflammatory microenvironment and improves gut health. Heliyon 2024, 10, e30594, doi:10.1016/j.heliyon.2024.e30594.
42. Jing, Y.; Wang, Z.; Cheng, W.; Fan, H.; Zheng, K.; Zheng, Y.; Wu, L. Structure Characterization and Treatment Effect of Zingiber officinale Polysaccharide on Dextran Sulfate Sodium-Induced Ulcerative Colitis. Foods 2025, 14, doi:10.3390/foods14050753.
43. CEN, L. Houttuynia cordata polysaccharides alleviate ulcerative colitis by restoring intestinal homeostasis; 2022; Volume 20, pp. 914-924.
44. Su, L.; Mao, C.; Wang, X.; Li, L.; Tong, H.; Mao, J. The Anti-colitis Effect of Schisandra chinensis Polysaccharide Is Associated With the Regulation of the Composition and Metabolism of Gut Microbiota; frontiersin.org: 2020.
45. Li, J.; Tao, W.; Zhou, W.; Xing, J.; Luo, M.; Lu, S.; Yang, Y. Dendrobium officinale polysaccharides alleviated DSS-induced ulcerative colitis in mice through remolding gut microbiota to regulate purine metabolism. Journal of Functional Foods 2024, 119, doi:10.1016/j.jff.2024.106336.
